# Transcriptome, metabolome, and inflammatory and oxidative properties of *Clostridium butyricum* CB1002 clinical strain and its isogenic mutant Δ*hbd*

**DOI:** 10.1128/aem.01106-25

**Published:** 2026-02-23

**Authors:** Lancelot Nicolas, Victoria Mesa, Laurent Ferraris, Johanne Delannoy, Thomas Cokelaer, Céline Chollet, Florence Castelli, Amal Zerrad-Saadi, Frédéric Barbut, Julio Aires

**Affiliations:** 1 Université Paris Cité, Inserm U1139 (FPRM) 555089, Paris, France; 2 Plate-forme Technologique Biomics, Institut Pasteur, Université Paris Cité 555089, Paris, France; 3 Laboratoire d'Etude du Métabolisme des Médicaments, CEA, Université Paris Saclay 27048, Gif-sur-Yvette, France; Washington University in St. Louis, St. Louis, Missouri, USA

**Keywords:** IL10, IL17, IL22, interferon gamma, metabolome, transcriptome, redox, *Clostridium butyricum*, necrotizing enterocolitis, NEC

## Abstract

*Clostridium butyricum* CB1002, a clinical strain associated with a fatal case of necrotizing enterocolitis (NEC), lost its enteropathogenicity in an animal model of NEC, after genetic inactivation of the *hbd* gene. In this study, we compared the transcriptomes and metabolomes of CB1002 and CB1002 Δ*hbd*. Additionally, we evaluated the inflammatory and oxidative properties of both strains *in vitro*. RNA-Seq was performed on a mid-exponential growth phase culture. Untargeted metabolomics was performed on bacterial culture supernatants. Human peripheral blood mononuclear cells were used to measure IL-10, IL-17, IL-22, and IFNγ. Caco-2 cell line was used to assess reactive oxygen species (ROS), superoxide anions (O_2_^•−^), and peroxynitrite compounds (ONOO^–^). A total of 670 genes were identified as being differentially expressed between CB1002 Δ*hbd* and CB1002 (*P* < 0.05). Sixty-one of these genes were associated with potential virulence mechanisms and included bacterial metabolism (*n* = 17), cell wall peptidoglycan (*n* = 19), chemotaxis and quorum sensing (*n* = 14), flagellar assembly (*n* = 2), and oxidative stress response pathways (*n* = 9). *In vitro,* we showed that IL-10 stimulation by bacterial debris from CB1002 Δ*hbd* was decreased compared with CB1002 (*P* < 0.05). There were no significant differences in the levels of ROS, O_2_^•−^, and ONOO^-^ levels between the different experimental conditions and strains. Our results provide new genetic insights into the potential molecular mechanisms linking *C. butyricum* and NEC. This work supports the hypothesis that the *hbd* deletion resulted in transcriptional alterations affecting bacterial virulence.

**IMPORTANCE:**

*Clostridium butyricum* is a potential opportunistic pathogen that has been associated with NEC. We compared the transcriptomic and the metabolomic profiles of a clinical strain associated with a fatal case of NEC and its Δ*hbd* isogenic mutant. The *hbd* gene encodes a β-hydroxybutyryl-CoA dehydrogenase involved in the initial steps of the central carbohydrate fermentation pathway, converting pyruvate into butyrate production. We identified significant transcriptional changes linked to potential virulence mechanisms, including bacterial metabolism and cell wall biosynthesis, chemotaxis, and the oxidative stress response. Using human cell models, we performed functional assays that evaluated immune and redox cellular responses and found that the *hbd* deletion had no effect on *in vitro* inflammatory or oxidative activity. Our findings offer novel insights into the genetic determinants of *C. butyricum* pathogenicity and their relevance to NEC pathogenesis. Our results support the hypothesis that the *hbd* deletion affects bacterial virulence by transcriptional reprogramming.

## INTRODUCTION

Necrotizing enterocolitis (NEC) is one of the most common neonatal intestinal diseases occurring in extremely preterm neonates (PNs) (1, 2). In high-income countries, the incidence of NEC ranges from 2% to 13%, depending on the geographic area and the reporting period (3). NEC is associated with a high mortality rate up to 30% as well as long-term complications, such as short bowel syndrome, growth delays, and neurodevelopmental delays in infants who survive surgical intervention (4). NEC is a multifactorial disease, whose pathogenesis remains unclear. Data on microbial dysbiosis have highlighted the impact of abnormal microbiota of the PN on NEC (5); however, the mechanistic understanding of the relationship between bacteria and NEC development is incomplete. No specific microorganism has been definitively linked to NEC. However, the most commonly proposed bacterial etiological agents of NEC belong to the *Enterobacteriaceae* and the *Clostridium* genera (6, 7). Concerning *Clostridium*, epidemiological studies, clinical signs, and animal models have reported the involvement of clostridia strains in the development of NEC, including *Clostridium butyricum* (7).

*C. butyricum* belongs to the *sensu stricto* cluster I of the genus *Clostridium*, a strictly anaerobic, Gram-positive, sporulating bacterium that is widely distributed in the environment and in the intestines of humans and animals (8). *C. butyricum* has been reported to be part of the gut microbiota in PN (9). However, intestinal colonization of PNs by *C. butyricum* has been associated with NEC (10–12). In a preterm piglet model, *C. butyricum* was significantly overrepresented in colonic mucosal samples from piglets with NEC (13). Smith et al. reported a correlation between *C. butyricum* presence and pneumatosis intestinalis in tissue samples from neonates with NEC (14).

Pneumatosis intestinalis and portal venous gas are pathognomonic radiographic signs of NEC and have been proposed to result from gas production due to bacterial fermentation metabolism. Using germ-free quails fed with a lactose diet and mono-associated with *C. butyricum*, animals developed cecal NEC-like lesions (15). In contrast, animals colonized with *Klebsiella pneumoniae* or *Clostridioides difficile* did not develop NEC-like lesions (15). Furthermore, animals colonized with *C. butyricum* CB1002 clinical strain showed significantly more frequent and severe intestinal NEC-like lesions than those colonized with CB1002 isogenic strain deleted in the *hbd* gene, which encodes a β-hydroxybutyryl-CoA dehydrogenase involved in the initial steps of the central carbohydrate fermentation pathway, converting pyruvate to butyrate production (16). Of note, inflammatory and redox analysis of the host-cellular response has not been performed.

No specific phenotypic or molecular marker can distinguish asymptomatic carriage of *C. butyricum* isolates. Efforts to identify the genes responsible for encoding specific virulence factors in this non-toxigenic species have been unsuccessful. However, possible non-specific virulence genes have been suggested based on a comparative genomics analysis (17, 18). To test the hypothesis that gene expression may contribute to virulence*,* we performed a transcriptomic comparison of the clinical *C. butyricum* 1002 and *C. butyricum* 1002 Δ*hbd* strains. In addition, we investigated the pro-inflammatory and redox properties of both strains *in vitro*.

## MATERIALS AND METHODS

### Bacterial strains and culture conditions

Strain *C. butyricum* CB1002 was isolated from the fecal samples of a fatal NEC case (19). CB1002 Δ*hbd* (HBD) is the isogenic strain of CB1002 WT, inactivated for butyrate production via homologous recombination of the *hbd* gene, which encodes a β-hydroxybutyryl-CoA dehydrogenase involved in butyrate fermentation (16). Strains were cultured on Columbia cysteine agar (Oxoid; Thermo Fisher Diagnostics SAS, Dardilly, France) supplemented with 5% sheep blood at 37°C for 72 h. Liquid cultures were grown overnight at 37°C in either 10 mL brain heart infusion (BHI) or Tryptone-glucose-yeast extract (TGY) broth. All cultures were incubated in an anaerobic chamber (Coy Laboratory Products Inc, Grass Lake, MI, USA) with a gas mixture of 90% N_2_, 5% H_2_, and 5% CO_2_.

### RNA extraction, rRNA depletion, and cDNA library preparation

For RNA extraction, CB1002 WT and CB1002 HBD cultures were grown overnight in 20 mL BHI broth at 37°C, then sub-cultured in 250 mL BHI broth to mid-exponential phase (OD600 = 1.1 ± 0.5). Cultures were harvested at 4°C and resuspended in 500 µL RNAlater (Invitrogen, Carlsbad, CA, USA), then stored at −80°C. RNA was extracted using the FastRNA Pro Blue kit (MP Biomedicals, Irvine, CA, USA) and bead-beater instrument, then purified with the ZYMO Direct-zol RNA Miniprep Plus kit (Zymo Research, Irvine, CA, USA). RNA concentration and purity were measured using the Qubit RNA HS assay kit and Qubit 2.0 fluorometer (Life Technologies, Grand Island, NY, USA). Integrity was confirmed using the Agilent 2100 Bioanalyzer (Santa Clara, CA, USA) based on an RNA integrity number (RIN) > 7. A total of 100 ng RNA was used for RNA-Seq library construction with the Illumina stranded Total RNA Ligation Ribo0+ kit, followed by ribosomal RNA depletion. Libraries were sequenced on an Illumina NextSeq 2000 (Illumina, Inc., San Diego, CA, USA) using single-read mode (50–75 base-length reads).

### RNA-Seq analysis

RNA-Seq analysis was performed using the Sequana RNA-Seq pipeline (v0.92.2; https://github.com/sequana/rnaseq) with default parameters, unless stated otherwise (20). The pipeline first checked ribosomal content (<5%) and read quality with FastQC v0.11.9 (http://www.bioinformatics.babraham.ac.uk/projects/fastqc/). Reads were then trimmed for adapters and low-quality bases using fastp v0.20.1 (21). The filtered reads were aligned to the CB1002 WT genome using Bowtie2 v2.4.5A D (22). Normalization corrected for technical biases, and differential analysis between HBD and WT groups was done using DESeq2 (23) with the rnadiff container from the Damona project (damona.readthedocs.io). Genes with an adjusted *P*-value < 0.05 were considered significant..

### CB1002 genome sequencing, assembly, and annotation

DNA extraction was performed on 24-h bacterial liquid cultures using the DNA easy UltraClean microbial kit (Qiagen, Hilden, Germany), following the manufacturer’s instructions. Paired-end libraries were prepared using the TruSeq DNA PCR-free sample prep kit (Illumina, Inc., San Diego, CA, USA) and sequenced on a HiSeq 2500 system (Illumina, Inc., San Diego, CA, USA) according to the manufacturer’s instructions. Genome sequences were assembled as previously reported (24). The genome assembly was annotated automatically using the MicroScope pipeline platform v3.14.3 (25).

### GO annotation and KEGG pathway analysis of differentially expressed genes

To predict metabolic pathways, differentially expressed genes (DEGs) were compared with the Kyoto Encyclopedia of Genes and Genomes (KEGG) and Clusters of Orthologous Groups (COG) databases for annotation and classification. Pathways were annotated using the KAAS-KEGG Automatic Annotation Server (https://www.genome.jp/kegg/kaas/), and gene families were characterized by COG functional category using eggNOG-mapper v5.0 software (26).

For GO and KEGG enrichment analysis, we used the “clusterProfiler” (version 4.10.1) package of R software to conduct GO function and KEGG pathway enrichment analysis on DEGs, particularly over-representation analysis (ORA) to evaluate associated pathways.

### Protein-protein interaction network

To investigate the inter-relationship of the DEGs, protein-protein interaction (PPI) network of target genes was retrieved using the online Search Tool for the Retrieval of Interacting Genes (STRING) database version 12 (27). We set the STRING database to include both experimentally verified interactions and predicted functional connections between proteins. It assigns scores to protein pairs, indicating the strength of their functional relationships. These STRING scores are derived from the gene co-occurrence, co-expression, and previous knowledge in the database. MCL clustering with inflation parameters was applied to find out the top three clusters. The minimum required interaction score was set to 0.4.

### Cell culture and isolation

All medium components were purchased from Gibco (Fisher Scientific Diagnostics SAS, Dardilly, France), unless otherwise stated. Cells were incubated for 24 h at 37°C, 95% air, 5% CO_2_, and 100% humidity. Human colon adenocarcinoma Caco-2 cells were cultured and maintained in Dulbecco’s Modified Eagle Medium (DMEM)-GlutaMax-I, with [+]4.5 g/L D-glucose, supplemented with 1% (v/v) non-essential amino acids, 10% (v/v) fetal bovine serum (FBS), 10 units/mL penicillin, and 10 μg/mL streptomycin, as previously reported (28). Peripheral blood mononuclear cells (PBMCs) were isolated from healthy adult donors’ blood, without pooling, using an Histopaque solution (Sigma-Aldrich, Inc., St. Louis, USA) (centrifugation 400 × *g*, 20 min, at room temperature). Cells were washed twice in Dulbecco’s Phosphate Buffered Saline (DPBS)−1×, centrifuged at room temperature (400 × *g*, 10 min), and resuspended in Roswell Park Memorial Institute (RPMI)-GlutaMax-I, supplemented with FBS (10% v/v), HEPES buffer solution (1% v/v), and penicillin/streptomycin (1% v/v).

### Bacteria and bacterial components for cellular assays

Bacteria and bacterial components were prepared using a 24-h TGY bacterial sub-culture (20 mL). After centrifugation (3,500 × rpm, 20 min, at 15°C), the culture supernatants were filtered (0.2-μm-pore-size filter; Millipore) and stored at –80°C for metabolomics analysis. The bacterial pellets were washed, resuspended in DPBS (1×), and divided into aliquots as follows: live bacteria, dead bacteria after boiling (100°C for 15 min); bacterial debris and bacterial cytoplasmic contents obtained after sonication (30 s, 15 s pause, 30 s) (Vibra-Cell, Bioblock Scientific, France) and centrifugation (13,000 rpm, 10 min, at 4°C). Bacteria and bacterial components were distributed into each well of the culture plates at a concentration of 1/20 (v/v).

### Cytokine assays

Twenty-four-well culture plates were coated for 1 h at 37°C under CO_2_ atmosphere (95% air, 5% CO_2_, and 100% humidity) with Ultra-LEAF Purified anti-human CD3 Antibody (BioLegend, San Diego, CA, USA) (2 μg/mL in RPMI). Then, PBMC cells (1 × 10^6^ cells/mL) were added to the plates and activated with Ultra-LEAF Purified anti-human CD28 Antibody (BioLegend, San Diego, CA, USA) (4 μg/mL in RPMI), and incubated for 24 h with live bacteria and bacterial components. After centrifugation, supernatants were collected and frozen at −20°C. IL-10, IL-17, IL-22, and IFNγ secretion were quantified by ELISA (R&D Systems Inc., Minneapolis, MN, USA), following the manufacturer’s instructions. The control consisted of PBMC cells that were activated using an anti-human CD3/CD28 antibody complex. The assays included three biological experiments with technical duplicates.

### Redox assay

Reactive oxygen species (ROS) production was assessed using the CM-H2DCFDA probe according to the manufacturer’s instructions (Invitrogen, Carlsbad, CA, USA). Briefly, CM-H2DCFDA probe was suspended in DMSO and diluted in Hanks’ Balanced Salt Solution (HBSS) at a final concentration of 5 µM. Caco-2 cells (1 × 10^6^ cells/well/plate) were incubated with the probe for 1 h (37°C, 5% CO_2_, and 100% humidity). Then, live bacteria and bacterial components were added for 30 min co-incubation in dye-free medium. The medium was then replaced with fresh dye-free medium, and measurements were performed using a microplate reader at Ex/Em = 492/518 wavelength (Enspire 2300 Multilabel Reader—PerkinElmer, Waltham, MA, USA). The control consisted of Caco-2 cells incubated with the CM-H2DCFDA probe and an exogenous inducer of oxidative stress, tert-butyl hydroperoxide. O₂^•–^ production levels by NADPH oxidase were measured as previously described and adapted for bacterial samples (29). ONOO^−^ production levels were measured using Peroxynitrites Assay Kit (Cell-based) ab233468 (Abcam, Cambridge, UK) according to the manufacturer’s instructions. The controls consisted of Caco-2 cells incubated with *Escherichia coli* K12 lipopolysaccharide at a final concentration of 1,000 ng/mL. Assays included three to five biological experiments with technical duplicates.

### Untargeted metabolomics

Culture supernatants were processed for metabolite extraction and analysis by the LEM facility (CEA Paris-Saclay, France), as previously described (30). Metabolites were extracted using methanol-assisted protein precipitation and analyzed via liquid chromatography-high-resolution mass spectrometry (LC-HRMS), combining reversed-phase (C18) and HILIC methods (excluding fatty acids and complex lipids). LC-HRMS was performed on an Ultimate 3000 system coupled to Exactive or Q-Exactive mass spectrometers (Thermo Fisher Diagnostics SAS, Dardilly, France), using electrospray ionization in both positive (C18) and negative (HILIC) modes. Metabolite annotation was done using an internal spectral database, and peak integration was manually reviewed. Peak areas were normalized to QC samples using the LOESS algorithm to correct for batch effects (31). Final data were expressed in arbitrary units, representing chromatographic peak areas per metabolite.

### Short-chain fatty acid analyses

Gas chromatography was used to measure acetate, propionate, and butyrate levels in the cell-free culture supernatant samples of the wild-type (WT) and Δ*hbd* mutant strains, as described previously (16).

### Statistical analysis

Experimental data were analyzed using GraphPad Prism 8.0.1 (La Jolla, CA, USA). Comparisons between CB1002 HBD and WT strains were performed using the unpaired, Welch-corrected Student’s *t*-test. Metabolite data were analyzed using both univariate and multivariate approaches in R (R Core Team, 2022; https://www.R-project.org). The relationship between gene expression and metabolites was tested using Spearman’s rank correlation coefficient. The R software package “corrplot” (Version 0.95) was used for correlation visualization (https://github.com/taiyun/corrplot). Statistical significance was set at *P* < 0.05.

## RESULTS AND DISCUSSION

### *De novo* transcriptome assembly

RNA-Seq produced high-quality data sets averaging 24 million reads per sample, with 93% of bases scoring above Q30, and 99% mapping to the CB1002 WT genome. Read normalization minimized false positives (Fig. S1), and a clear separation was observed between the CB1002 HBD and WT samples (Fig. S2). To analyze transcriptomic changes, the CB1002 WT genome was sequenced and annotated, revealing a genome size of 4.57 Mb with 28.52% G+C content, which is consistent with the reference strain VPI3266^T^. A total of 4,198 coding sequences were annotated.

### Identification of DEGs

Of the annotated genes, 2,054 exhibited differential expression, 670 of which were significantly different (*P* < 0.05). This included 328 upregulated genes and 342 downregulated genes. KEGG enrichment analysis revealed that these genes were involved in metabolic and biosynthetic pathways (40%), ABC transporters (3%), two-component systems (2%), quorum sensing (1%), and chemotaxis and motility (1%) (Fig. S3). According to the COG enrichment analysis, most of the matched genes were associated with unknown functions (29%), replication and transcription and translation (21%), carbohydrate metabolism (7%), energy production (5%), and ion transport (6%) (Fig. S4).

To investigate the observed loss of enteropathogenicity observed in the quail NEC model following the *hbd* mutation (16), we specifically focused on potential virulence genes that were differentially expressed between CB1002 HBD and WT. These included genes associated with bacterial chemotaxis, flagellar and pilus assembly, quorum sensing, metabolism, cell wall synthesis, and oxidative stress response. Table 1 lists the 61 genes of interest that we identified.

**TABLE 1:** Differentially expressed genes between CB1002 HBD and CB1002 WT (*P*-value < 0.05)

Locus	Gene ID^*a*^	Log2 fold change	KEGG	Function	Class	Category	Subcategory
CBUT_v1_50196	hbd	−7.01	K00074	3-Hydroxybutyryl-CoA dehydrogenase		Metabolism	Carbohydrate metabolism
CBUT_v1_50099	ptb	−3.87	K00634	Phosphate butyryltransferase			
CBUT_v1_50100	buk	−3.98	K00929	Butyrate kinase			
CBUT_v1_130138	penP_1	−1.24	K17836	Penicillin-binding protein	Lac I	Metabolism	Biosynthesis of other secondary metabolites
CBUT_v1_170135	penP_2	2.56	K17836	Penicillin-binding protein			
CBUT_v1_130585	gloA	0.71	K01759	Lactoylglutathione lyase			Carbohydrate metabolism
CBUT_v1_120031	lacZ	−3.1	K01190	Beta-galactosidase			
CBUT_v1_130150	ldh_1	1.01	K00016	L-lactate dehydrogenase			
CBUT_v1_370026	ldh_2	0.48	K00016	L-lactate dehydrogenase			
CBUT_v1_230017	galT	1.09	K00965	Galactose-1-phosphate uridyltransferase			
CBUT_v1_230019	galK	0.85	K00849	Galactose-1-phosphate uridyltransferase			
CBUT_v1_230022	agaA	2.98	K07407	Alpha-galactosidase agaa			
CBUT_v1_230064	cbgA	−2.35	K01190	Beta-galactosidase			
CBUT_v1_50101	ilvC	−3.03	K00053	Acetohydroxy-acid isomeroreductase (NADP-dependent)			Global and overview maps
CBUT_v1_50118	*ilvB*	-1.2	K01652	Acetohydroxy-acid synthase (large subunit)			
CBUT_v1_140080	*comB*	0.53	K05979	Putative 2-phosphosulfolactate phosphatase			Energy metabolism
CBUT_v1_230055	*galR_1*	-0.9	K02529	Transcriptional regulators of the laci family		Genetic information processing	Transcription factors
CBUT_v1_230016	*galR_2*	1.7	K02529	Transcriptional regulators of the laci family			
CBUT_v1_130571	*comEC*	0.8	K02238	Metallo beta-lactamase family protein		Cellular processes	Transporters
CBUT_v1_130592	*lctP*	-3.17	K03303	L-lactate permease			
CBUT_v1_130450	*oppB*	-3.31	K15581	Oligopeptide transporter subunit			
CBUT_v1_130451	*oppC*	-3.29	K15582	Oligopeptide transporter subunit			
CBUT_v1_130452	*oppD_1*	-3.28	K15583	Oligopeptide transporter subunit			
CBUT_v1_130453	*oppD_2*	-2.94	K10823	Oligopeptide transporter subunit			
CBUT_v1_130454	*oppA*	-3.35	K15580	Oligopeptide transporter subunit			
CBUT_v1_130462	*oppF*	1.14	K02032	Oligopeptide transporter subunit			
CBUT_v1_230018	*galE*	0.92	K01784	UDP-glucose 4-epimerase	Peptidoglycan biosynthesis	Cellular processes	Cellular community
CBUT_v1_370041	*argC*	-1.41	K00145	N-acetyl-gamma-glutamyl-phosphate reductase			
CBUT_v1_370042	argJ	−2.09	K00620	Amino-acid N-acetyltransferase			
CBUT_v1_370043	argB	−1.62	K00930	Acetylglutamate kinase			
CBUT_v1_370044	argD	−1.6	K00821	Acetylornithine aminotransferase			
CBUT_v1_370045	argF	−1.37	K00611	Ornithine carbamoyltransferase			
CBUT_v1_190452	glpK	−1.77	K00864	Glycerol kinase			
CBUT_v1_190453	glpF	−1.73	K02440	Glycerol uptake facilitator			
CBUT_v1_170230	licA	−2.02	K02759	Cellobiose PTS system EIIA component			
CBUT_v1_180113	vanR	−3.81	K18349	Response regulator ompr family			Transcription factors
CBUT_v1_140030	glmU	−0.81	K04042	Fused N-acetyl glucosamine-1-phosphate uridyltransferase		Metabolism	
CBUT_v1_170187	murA	−1.72	K00790	UDP-N-acetylglucosamine 1-carboxyvinyltransferase			Carbohydrate metabolism
CBUT_v1_170078	ddlB	0.55	K01921	D-alanine:D-alanine ligase			Glycan biosynthesis and metabolism
CBUT_v1_130478	cheY	−1.59	K03413	Regulator of chemotaxis and motility			
CBUT_v1_130866	cheX	0.78	K03409	Putative chemotaxis phosphatase protein chex			
CBUT_v1_130921	mcpB_1	4.55	K03406	Putative chemotaxis protein transducer	Chemotaxis	Cellular processes	Cell motility
CBUT_v1_130962	mcpB_2	1.07	K03406	Methyl-accepting chemotaxis protein mcpb with signaling domain			
CBUT_v1_140076	mcpB_3	−3.05	K03406	Methyl-accepting chemotaxis protein mcpb with signaling domain	Chemotaxis	Cellular processes	Cell motility
CBUT_v1_190141	cheV	0.48	K03415	Putative chemotaxis transducer			
CBUT_v1_190458	mcpB_4	−0.93	K03406	Methyl-accepting chemotaxis sensory transducer with signaling domain			
CBUT_v1_190541	mcpB_5	2.14	K03406	Methyl-accepting chemotaxis protein with preiplasmic sensor-like domain and signaling domain			
CBUT_v1_280039	mcpB_6	3.09	K03406	Putative chemotaxis protein with accepting receptor and signaling domain			
CBUT_v1_290003	mcpB_7	1.45	K03406	Putative chemotaxis protein with preiplasmic sensor-like domain and signaling domain			
CBUT_v1_300126	mcpB_8	−2.46	K03406	Putative chemotaxis protein with preiplasmic sensor-like domain and signaling domain			
CBUT_v1_300127	mcpB_9	3.51	K03406	Putative chemotaxis protein with accepting receptor and signaling domain			
CBUT_v1_130250	pilT	−1.68	K02669	Putative chemotaxis protein with accepting receptor and signaling domain			
CBUT_v1_130053	pilB	−2.03	K02652	Type IV pilus assembly protein			
CBUT_v1_50211	fliB	0.61	K18475	Flagellar protein flib	Flagellar	Organismal systems	Signaling and cellular processes
CBUT_v1_120010	motA	−1.02	K02556	Chemotaxis protein			
CBUT_v1_130917	nirA_1	2.54	K00366	Ferredoxin-nitrite reductase	Redox	Metabolism	Energy metabolism
CBUT_v1_150011	nirA_2	−0.72	K00366	Ferredoxin-nitrite reductase			
CBUT_v1_50016	trxB	1.07	K00384	Thioredoxin reductase			Metabolism of other amino acids
CBUT_v1_50018	grxC	1.83	K03676	Putative glutaredoxin		Genetic information processing	Transcription factors
CBUT_v1_130184	trxA	−1.16	K03671	Thioredoxin		Organismal systems	Immune system
CBUT_v1_330030	sodA	0.63	K04564	Superoxide dismutase like		Environmental information processing	Signal transduction
CBUT_v1_250285	bcp	1.04	K03564	Thioredoxin-dependent peroxiredoxin		Unknown function	Unknown function

^*a*^ Based on automatic annotation through MaGe MicroScope platform (v3.17.3).

### Bacterial chemotaxis, flagellar, and pilus assembly-associated DEGs

Of the 61 DEGs, 14 belong to the chemotaxis-related KEGG classes, including *cheY*, *cheX*, *cheV*, nine *mcpB* genes, *pilT*, and *pilB* (Table 1). In CB1002 HBD, *cheY* was downregulated, while *cheX* and *cheV* were upregulated. The methyl-accepting chemotaxis McpB proteins interact with the CheA/CheY histidine protein kinases two-component signaling system, which controls flagellar movement in response to repellents. *cheX* and *cheV,* which encode coupling proteins*,* modulate CheY activity and affect motility (32). The flagellar assembly genes *motA* and *fliB* were also repressed in CB1002 HBD (Table 1). These data are consistent with the reduced swimming and swarming motility previously observed in CB1002 HBD compared with WT (14). Although not statistically significant (*P* > 0.05), 32 other flagellar assembly genes showed differential expression (Table S1). Additionally, *pilB* and *pilT* were repressed in CB1002 HBD. PilB is an ATPase involved in type IV pilus assembly, and PilT provides the power for pilus retraction, which is required for twitching motility (33). These findings are consistent with earlier observations of reduced twitching motility in CB1002 HBD (16). Chemotaxis and motility (flagella and pili) are tightly linked and play multiple roles in bacterial behavior, including adhesion, biofilm formation, autoaggregation, and host interactions. All of these processes are linked to virulence (34).

### Quorum sensing-associated DEGs

As shown in Table 1, we found that oppA, oppB, oppC, and oppD were downregulated in CB1002 HBD. These genes form an operon that encodes membrane-associated proteins of an ATP-binding cassette transporter (35). A variety of roles have been described for the bacterial Opp systems. In particular, they are involved in short peptide transport, including amino acid uptake and peptide-mediated signaling, through RRNPP type quorum sensing systems that regulate virulence gene expression (35). In addition, expression of the *argCJBDF* operon was repressed in CB1002 HBD (Table 1). *agrD* encodes a precursor peptide that is processed by AgrB into an autoinducing peptide (AIP), which activates the sensor kinase AgrC and initiates a response regulator cascade for gene expression (35). The Agr system also plays a fundamental role in quorum sensing, regulating the spatiotemporal expression of virulence factors, metabolic flexibility, and survival (36, 37). In *C. difficile*, the activation of *argD* and the downstream genes *argB* and *argC* may participate in toxin production, flagellar biosynthesis, and cyclic diguanylate signaling (36, 37). Several other quorum sensing-related genes (*dppD*, *ciaR*, *ciaH*, and nisin biosynthesis-related genes) were differentially expressed but did not reach statistical significance (Table S1).

### Metabolism-associated DEGs

We confirmed that the *hbd* gene was significantly repressed in CB1002 HBD, as were the *ptb* and *buk* genes. The *ptb* gene encodes a phosphotransbutyrylase, and the *buk* gene encodes a butyrate kinase. These are the enzymes involved in the production of butyrate (38) (Table 1). Several sugar metabolism genes were also downregulated, including *lacZ*, *cbgA*, *agaA*, *ilvC, ilvB*, and *lctP*. LacZ, CbgA, and AgaA are glucosidases involved in sugar catabolism. *ilvB* and *ilvC* encode acetohydroxy acid synthase (IlvB) and ketoacid reductoisomerase (IlvC), respectively, which are involved in the biosynthesis of branched-chain amino acids (BCAAs). The *hbd* mutant may shift to use BCAAs as a redox-neutral overflow or for biosynthesis due to carbon/nitrogen imbalance. *lctP* encodes a lactate permease, whose repression suggests a limitation in lactate uptake. It is likely that CB1002 HBD downregulates certain sugar utilization and biosynthesis genes to avoid overloading its compromised fermentation network and maintain energy/redox balance. Virulence-related functions, such as galactosidase activity, lactate, and amino acid metabolism, have been directly and indirectly linked to pathogenicity (39–42).

In contrast, *galR*, *galTKE*, *glpKF*, and *ldh-1* were upregulated. Due to the *hbd* mutation, which blocks the butyrate pathway and impairs NADH oxidation, the upregulation of *ldh-1* may represent a compensatory response to restore redox balance. The *galTKE* operon, which is regulated by the GalR regulator, encodes the enzymes that are responsible for the catabolizing galactose via the Leloir pathway. This allows glucose-1-phosphate to enter glycolysis and be oxidized to pyruvate, enabling bacteria to survive and proliferate using alternative energy sources. This is consistent with the overexpression of the glycerol kinase GlpK and the glycerol uptake facilitator GlpF, which suggests a potential shift toward 1,3-propanediol production. Taken together, these data suggest a redirection of the bacterial metabolism affecting its biological properties and virulence.

### Bacterial cell wall-associated DEGs

The genes involved in the peptidoglycan biosynthesis were significantly differentially expressed in the CB1002 HBD strain (Table 1). *glmU* and *murA* were repressed in CB1002 HBD. These genes encode an N-acetylglucosamine-1-phosphate uridyltransferase and a UDP-N-acetylglucosamine-1-carboxyvinyltransferase, respectively. They are involved in the cytoplasmic steps of peptidoglycan UDP-N-acetylglucosamine biosynthesis (43). The *ddlB* gene, encoding a D-alanine ligase that catalyzes the incorporation of D-alanine into the bacterial cell wall lipoteichoic acids, was overexpressed in the CB1002 HBD strain (43). In *C. butyricum*, D-alanylation increases the hydrophobicity of the cell wall (44). Previously, we have reported that CB1002 HBD has higher surface hydrophobicity than CB1002 WT (16). The genes *penP* and *vanR,* encoding for a putative penicillin-binding protein and a putative cytoplasmic response regulator of the two-component system VanSR, were also differentially expressed in CB1002 HBD (Table 1). PenP and VanSR are associated with peptidoglycan modification (45). Interestingly, *comEC* and *comB*, which encode proteins involved in bacterial competence, were overexpressed in the mutant, suggesting possible cell wall modifications (46, 47). These modifications may contribute to changes in bacterial virulence through adhesion, biofilm formation, stress response, and resistance to cationic antimicrobial peptides (43). Of note, the *dltA*, *dltC*, *dltD,* and *vanY* were differentially expressed, though not significantly (Table S1).

### Oxidative stress-associated DEGs

Oxidative stress-related genes (*sodA*, *nirA_1*, and *ldh*) were significantly overexpressed in the CB1002 HBD strain compared with the WT strain (Table 1). *sodA* encodes a superoxide dismutase that detoxifies superoxide and works with catalase to scavenge ROS (48). *nirA* enables denitrification under nitrosative stress (49), and *ldh* supports redox balance (39). The *hbd* mutant, which is unable to oxidize NADH via butyrate synthesis, may shift to a redox compensation mode. These genes have been associated with increased survival and virulence under oxidative stress (50). Conversely, *trxB* and *bcp* were upregulated, while *trxA* was downregulated. *trxA* encodes an electron carrier of the thioredoxin system that donates electrons to reduce disulfide bonds in proteins. *trxB* encodes a thioredoxin reductase that regenerates reduced thioredoxin using NADPH (51). This expression pattern of the thioredoxin system may indicate that the bacteria are redirecting NADPH and defending against oxidative stress. This is consistent with the upregulation of *bcp,* which encodes a peroxiredoxin that detoxifies peroxides. It is also consistent with *sodA* expression, indicating that the bacteria is defending against both superoxide and peroxides. *gloA*, which encodes glyoxalase I for detoxifying the byproduct of central carbon metabolism, methylglyoxal, was also overexpressed (52). In the CB1002 HBD mutant strain, where butyrate production is blocked, causing pyruvate and upstream intermediates to accumulate. Overexpression of *gloA* helps prevent carbon-induced bacterial viability loss by detoxifying methylglyoxal. These systems are also involved in resistance to stress (51).

### Protein-protein network analysis

**Fig 1:**
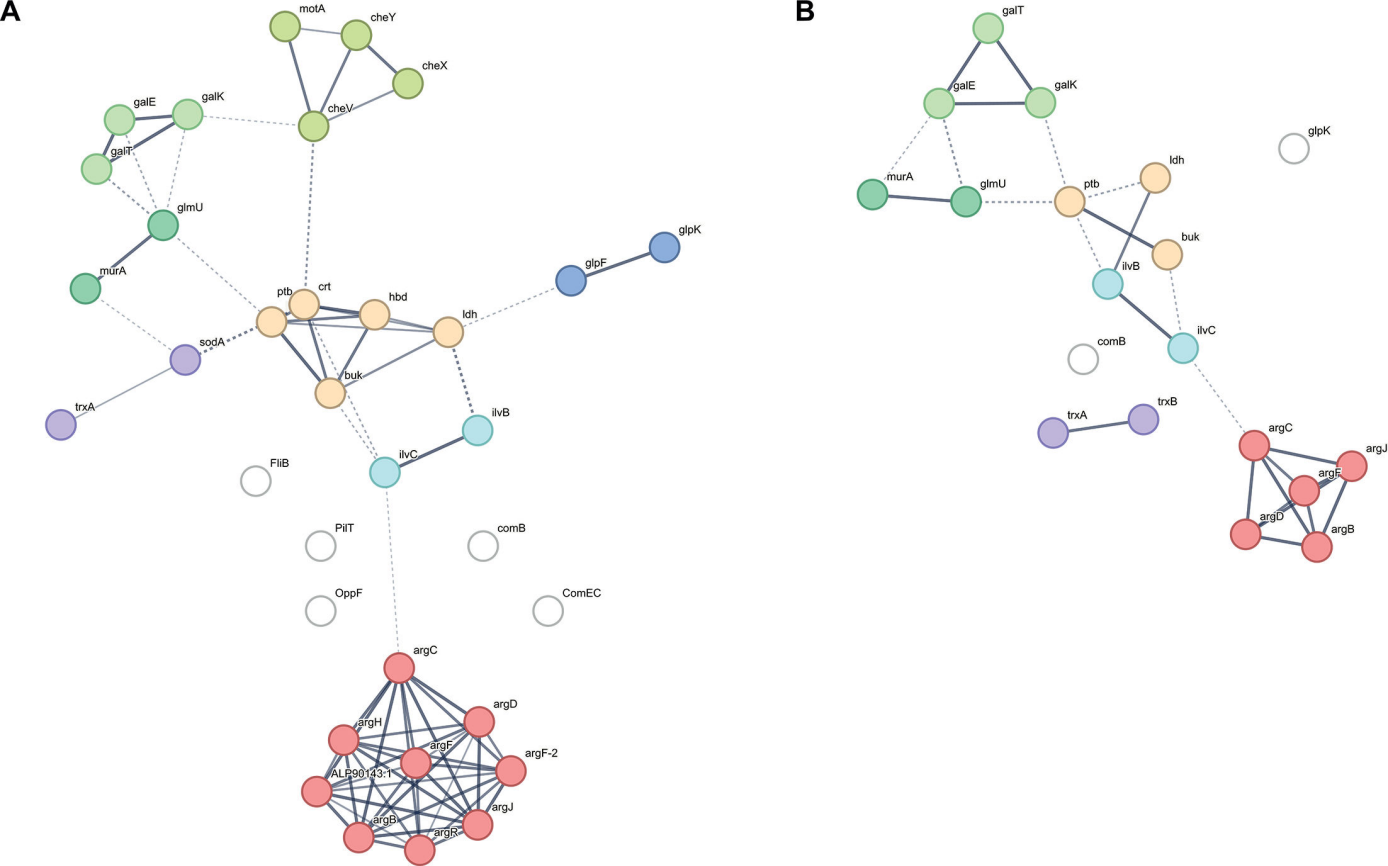
Protein-protein interaction network using the reference genomes of *C. butyricum* (=DSM 10702^T^) (**A**) and *C. neonatale* ATCC BAA-265^T^ (=CN250.09) (**B**) from the STRING database. The molecular network represents proteins depicted as nodes and their associations as edges. Each node corresponds to a single-gene protein product, while edges signify protein-protein interactions. The thickness of the lines indicates the level of confidence in the association. Different colors display various functional clusters. Colors are based on MCL clustering. Clusters are identified and grouped by color.

We analyzed the interactions among the 61 DEGs of interest. The interaction network for *C. butyricum* comprised 29 nodes and revealed eight clusters (Fig. 1A), including arginine, butyrate, galactose metabolism, bacterial chemotaxis, amino sugar biosynthesis (valine, leucine, and isoleucine biosynthesis), glycerol dehydrogenase and carbohydrate kinase, and oxidative stress-related proteins. We also compared the PPI networks of *C. butyricum* and *Clostridium neonatale. C. neonatale* is the closest species relative to *C. butyrium* within the genus *Clostridium sensu stricto* (cluster I) (53, 54). Additionally, *C. neonatale* has previously been isolated from NEC cases (55). Similar to *C. butyricum*, deletion of the *hbd* gene in the *C. neonatale* 250.09 clinical strain abolished enteropathogenicity in the quail NEC animal model (16). Analysis of the *C. neonatale* PPI network identified 19 nodes with clusters similar to those of *C. butyricum,* including valine, leucine, and isoleucine biosynthesis, galactose and butyrate metabolism, and oxidative stress-related proteins (Fig. 1B). Thirty-three proteins overlapped between the two species. The smaller *C. neonatale* network compared with *C. butyricum* suggests species-specific differences. However, the shared protein networks point to conserved pathways, which aligns with their similar enteropathogenic behavior in the quail NEC model (16).

### Gene expression correlations

**Fig 2:**
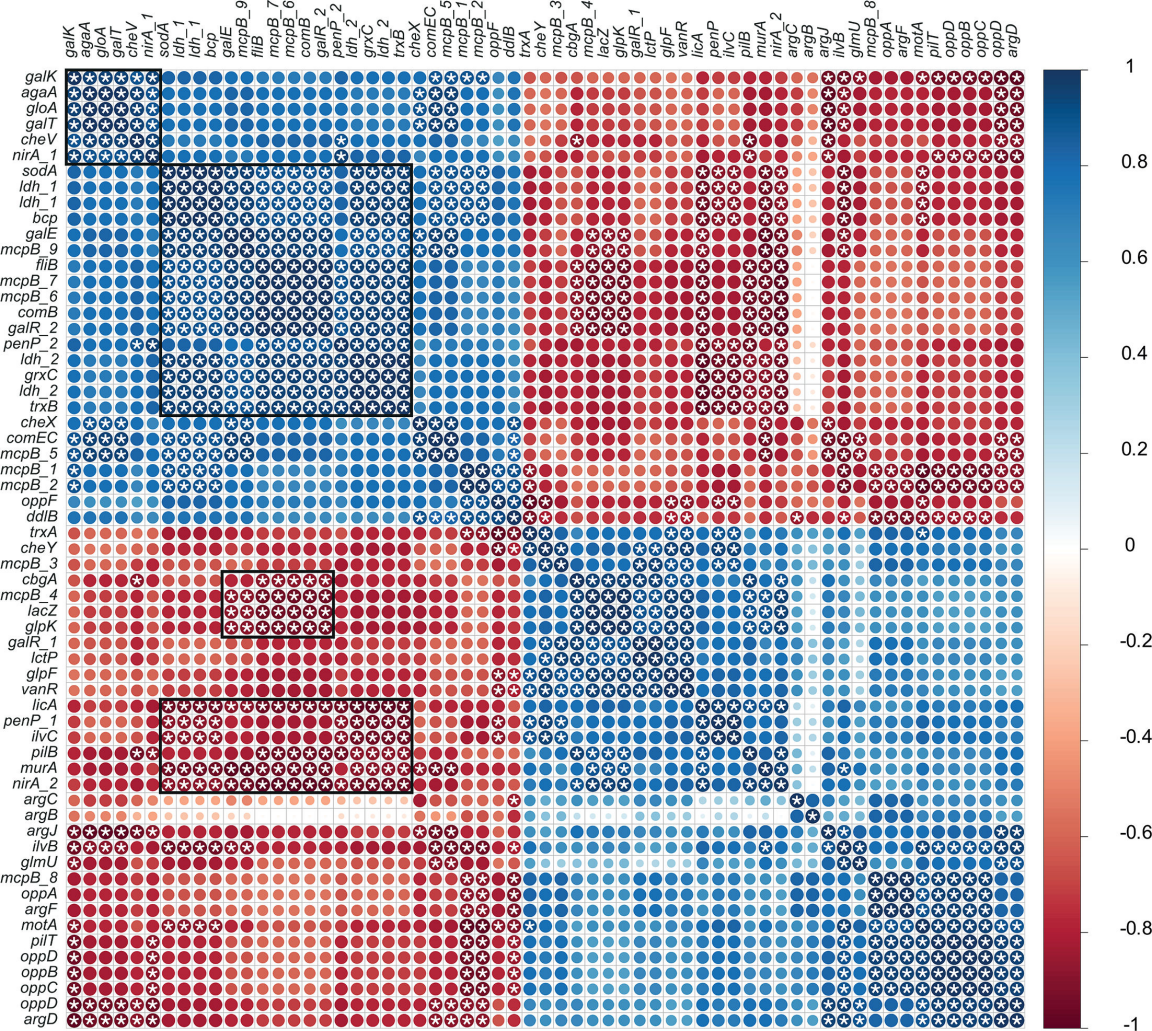
Spearman correlation matrix between differential gene expression. Scale indicates positive (blue) and negative (red) correlations. Yellow frames indicate examples of clusters. * indicate significant correlations (*P*-value < 0.05).

Figure 2 highlights distinct gene clusters whose expression was significantly altered following *hbd* deletion, suggesting disruption of normal metabolism. Overexpression of *galE*, *galR*, *galK*, *galT*, *agaA*, *sodA*, *ldh*, *bcp*, *mcpB*, *fliB*, *comB*, *grxC*, and *nirA* in CB1002 HBD suggests a shift in carbon flux, changes in cell wall composition, and increased use of alternative sugars and electron acceptors. Upregulation of *nirA* indicates an adjustment in nitrogen metabolism, possibly involving nitrate. This metabolic reprogramming may increase toxic byproduct formation, as reflected by coexpression of s*odA*, *bcp*, *grxC*, and *trxB*, which in turn may trigger *gloA*-mediated detoxification. Correlated expression of the *opp* operon, *arg* operon, *motA*, *pilT*, and *mcpB* points to a coordinated response to nutrient stress, enhancing nutrient uptake and environmental sensing involving quorum sensing. Upregulation of *mcpB*, *fliB*, and *che* genes supports a modification of chemotactic response to unfavorable conditions caused by the loss of the β-hydroxybutyryl-CoA dehydrogenase function. Notably, *gloA*, *nirA*, and *cheV* are also regulated by general stress-response pathways (51).

### Gene expression and metabolite correlations

**Fig 3:**
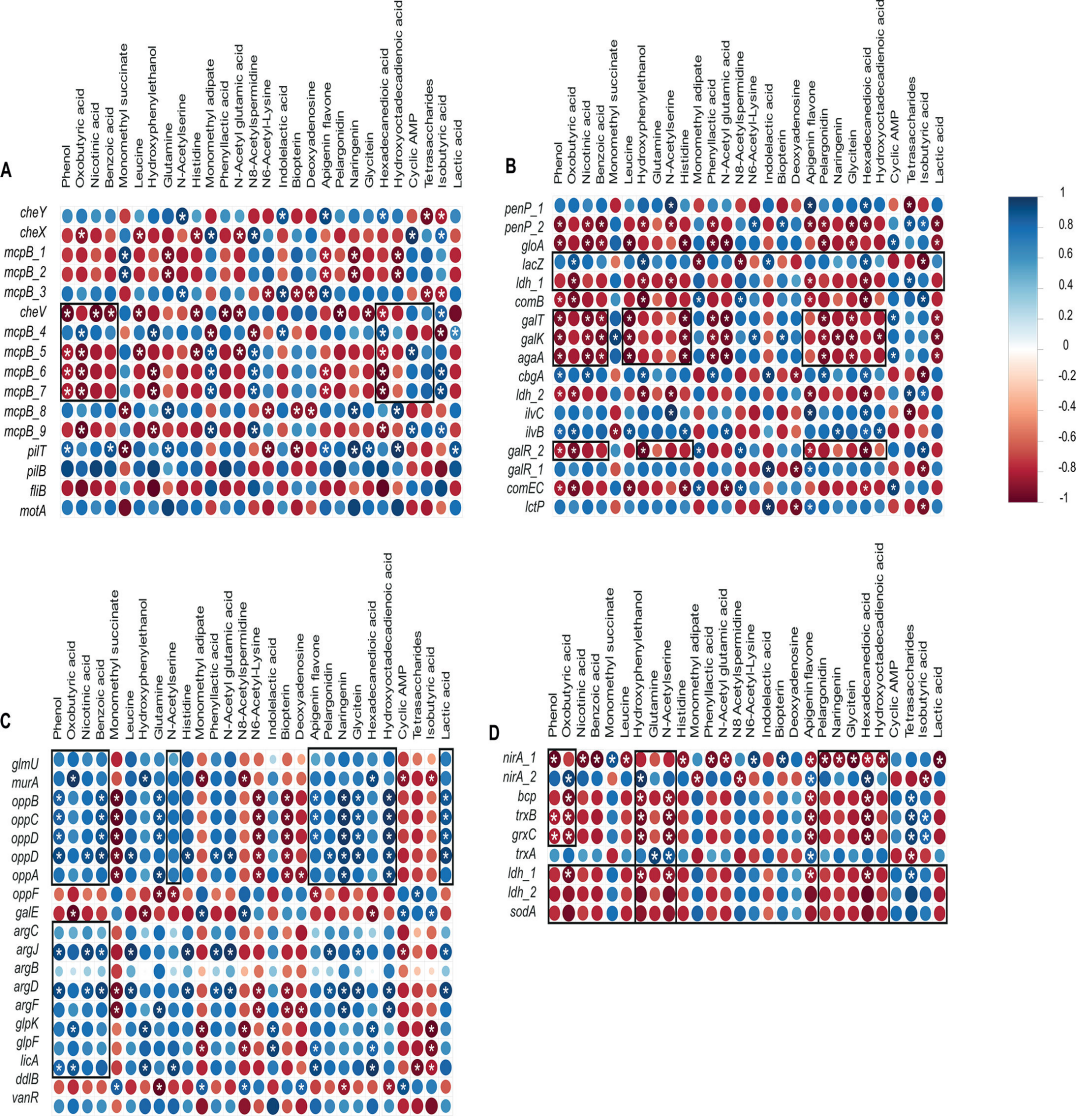
Spearman correlation matrix between metabolites and differential gene expression associated with chemotaxis (**A**), Lac family (**B**), peptidoglycan biosynthesis and quorum sensing (**C**), and oxidative stress (**D**). Scale indicates positive (blue) and negative (red) correlations. Yellow frames indicate examples of clusters. * indicate significant correlations (*P*-value < 0.05).

We confirmed that the culture supernatant of CB1002 HBD contained no detectable butyrate and produced acetate and propionate at levels similar to those previously reported for CB1002 WT (14). A total of 28 metabolites were identified through non-targeted metabolomics analysis of the same culture supernatant used for RNA-Seq analysis (Table S2). Figure 3 illustrates some of the correlations identified between metabolites and gene expression. Consistent with the overexpression of *galE*, *galR*, *galK*, *galT*, *agaA*, *sodA*, *ldh*, *bcp*, *mcpB*, *fliB*, *comB*, *grxC*, and *nirA*, we identified metabolites associated with a metabolic shift in CB1002 HBD. CB1002 HBD had higher levels of lactic acid, acetoin, oxobutyric acid, and sugars, which are associated with fermentation flux products. This is indicative of pyruvate overflow and shunting to multiple pathways (Table S2). We also identified indole lactic acid and phenyl lactic acid, derivatives of tryptophan and phenylalanine, respectively. These are associated with the degradation of amino acids during overflow metabolism under anaerobic and fermentative conditions. Notably, indole-lactic acid and phenyl-lactic acid have been reported to act on intestinal epithelial and immune cells, where they can signal through receptors, such as the aryl hydrocarbon receptor to dampen NF-κB-dependent pro-inflammatory cytokine production, enhance epithelial barrier function, and promote anti-inflammatory immune phenotypes (56). Thus, the accumulation of these metabolites in CB1002 HBD supernatant suggests that, *in vivo*, this strain may expose intestinal tissues to a qualitatively different metabolite profile with the potential to modulate epithelial responses and the local mucosal immune environment. The presence of the fatty acid derivative hexadecanedioic acid suggests a shift in lipid metabolism that may be associated with membrane remodeling, oxidative stress, or carbon overflow in fatty acid oxidation processes. The presence of glutamine, N-acetylserine, indole lactic acid, and phenyl lactic acid suggests rewiring of sulfur metabolism or oxidative stress. Therefore, in the context of a metabolic shift, intermediate byproducts accumulate, leading to stress responses. This is consistent with the identification of metabolites associated with nucleotide and polyamine-related metabolites, such as deoxyadenosine, cAMP, N8-acetylspermidine, and N6-acetyllysine, which are associated with signaling and cell stress response. This agrees with the overexpression of *sodA, bcp, grxC,* and *trxB*, and *gloA*, as well as increased oxidative stress and redox imbalance. Meanwhile, the identification of the following flavonoids—apigenin, genistein, pelargonidin, naringenin, and glycitein—may reflect mechanisms that help bacteria adapt to bacterial metabolic and environmental changes. This is consistent with the differential expression of genes that reflect chemotaxis, flagellar, and pilus processes.

In terms of gene-metabolite function associations, based on the KEGG MAPPER database and the KEGG compound ID, we confirmed that the identified metabolites were associated with map01100_Metabolic pathways (*n* = 23 metabolites), map01110_Biosynthesis of secondary metabolites (*n* = 14), map01120_Microbial metabolism in diverse environments (*n* = 8), map01230_Biosynthesis of amino acids (*n* = 6), map00941_Flavonoid biosynthesis (*n* = 3) and map00942_Isoflavonoid biosynthesis (*n* = 4), map01240_Biosynthesis of cofactors (*n* = 3), and map00230_Purine metabolism (*n* = 3).

### Inflammatory property analysis

**Fig 4:**
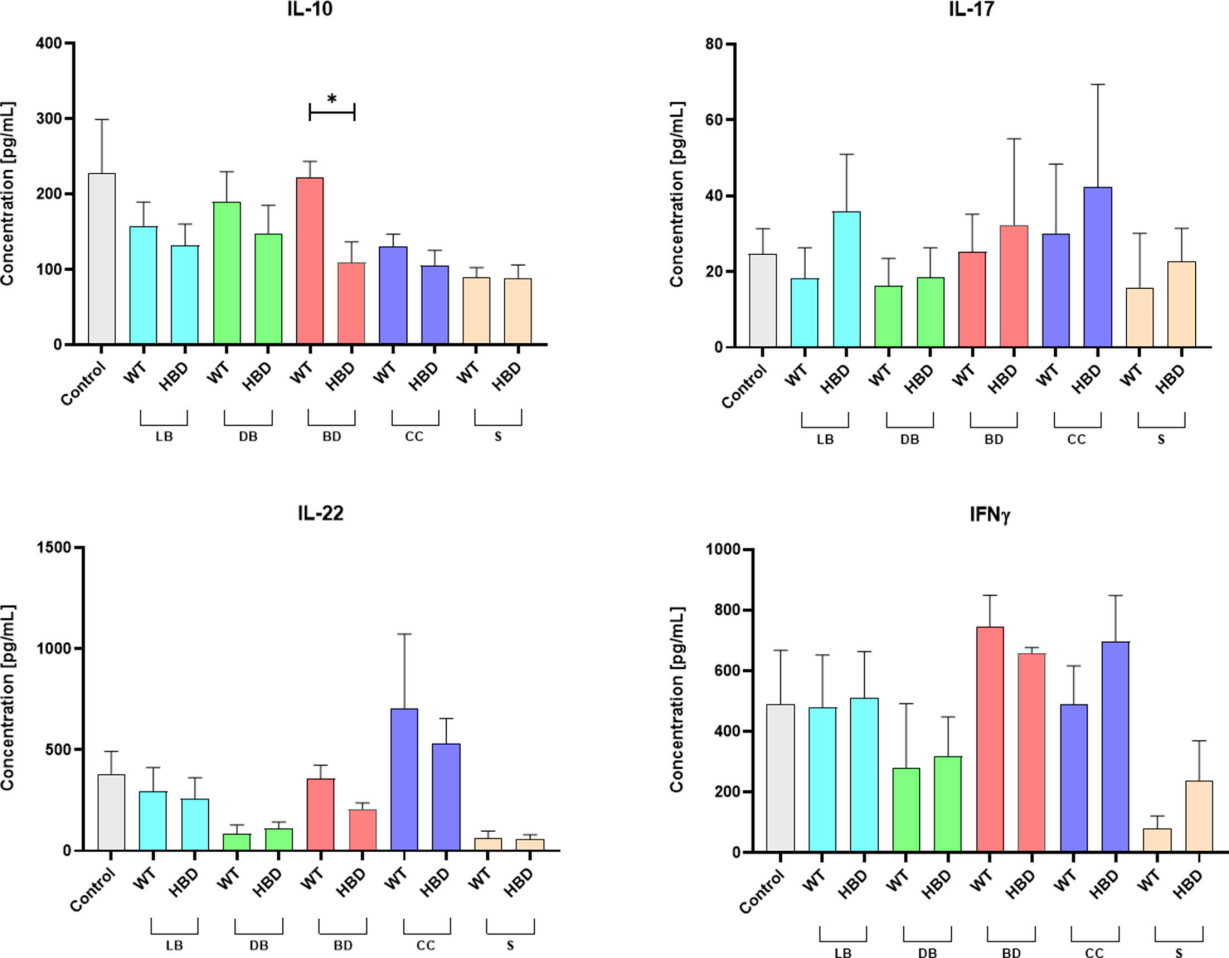
Dosage of interleukins IL-10, IL-17, IL-22, and interferon-γ (IFNγ) in PBMC culture supernatants after 24-h co-incubation with CB1002 HBD and WT bacterial treatments: LB, live bacteria; DB, dead bacteria (heat-treated); BD, bacterial debris; CC, cytoplasmic content; S, bacterial culture filtered supernatant. Results are displayed in absolute concentration values; means ± SEM. Statistical significance, *P-value < 0.05.

Intestinal inflammation has been identified as a significant contributing factor to the development of NEC (1, 2). Furthermore, bacterial involvement in local inflammation has been observed in NEC. In this study, we investigated the *in vitro* immunostimulatory properties of CB1002 strains on PBMCs. The levels of various cytokines, including IL-10, IL-17, IL-22, and IFNγ, in the culture supernatant of PBMCs after co-incubation with CB1002 HBD and WT live bacteria and bacterial components were not statistically significant, except for IL-10 production, which was significantly lower in the presence of bacterial debris from CB1002 HBD (Fig. 4). This finding suggests that the *hbd* deletion may alter bacterial cellular stimulation. PBMC cells from the control condition produce a higher concentration of IL-10 compared with the other experiment conditions, without statistical difference.

### Redox stress analysis

**Fig 5:**
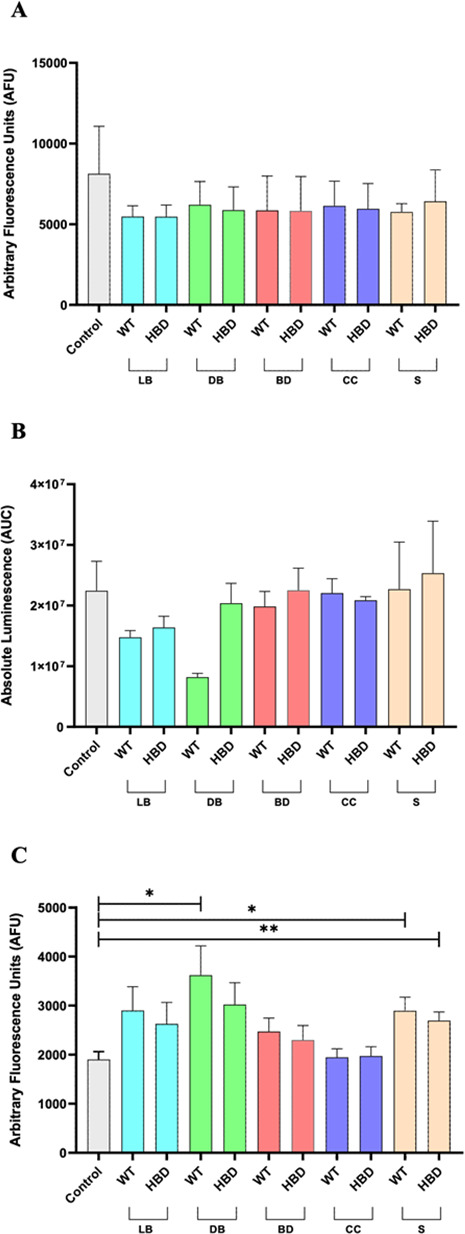
Dosage of global ROS (**A**), superoxide anions (O₂^•–^) (**B**), and peroxynitrites (ONOO^−^) (**C**) in Caco-2 culture supernatants after 30-min co-incubation with CB1002 HBD and WT bacterial treatments: LB, live bacteria; DB, dead bacteria (heat-treated); BD, bacterial debris; CC, cytoplasmic content; S, bacterial culture filtered supernatant. Results are displayed in arbitrary fluorescence units (AFU) or absolute luminescence area-under the ROC curve (AUC); means ± SEM. Statistical significance: *P-value < 0.05; **P-value < 0.001.

Oxidative stress has also been described as a contributing factor to NEC development (1, 2). Therefore, we analyzed the production levels of ROS, O₂^•–^, and ONOO^–^ production in Caco-2 cells after stimulation with the different bacteria samples. No statistically significant differences in total ROS, O₂^•–^, or ONOO⁻ levels were observed between CB1002 HBD and CB1002 WT under any of the conditions tested (Fig. 5). Thus, the butyrate-deficient mutant does not appear to differentially contribute to epithelial redox activation relative to the WT strain in the Caco-2 model. When compared with their respective unstimulated controls, however, all bacterial conditions tended to show a reduction in bulk ROS together with a concomitant increase in ONOO⁻, with significant ONOO⁻ increases detected for the WT DB, WT S, and HBD S conditions (Fig. 5A and C). These findings indicate that exposure to CB1002 preparations is associated with a redistribution of reactive species from classical ROS toward reactive nitrogen species, while the overall pattern of redox modulation remains similar between the WT and HBD strains in this epithelial model. Although the ONOO⁻ increase observed here was modest and comparable between CB1002 WT and HBD, a similar shift from ROS to peroxynitrite in an immature, inflamed intestine could still amplify nitrosative injury, promote enterocyte apoptosis, and impair barrier function even without large changes in bulk ROS. In this sense, CB1002 strains may not differ in how they modulate epithelial redox pathways relative to each other, but they do engage host redox signaling in ways that, in a susceptible neonatal gut, could intersect with established NO/iNOS–peroxynitrite-driven mechanisms of NEC (57).

### Limitations

One limitation of our study is that the RNA-Seq analysis identified less than 50% of the expressed genes from the 4,192 annotated genes in the sequenced CB1002 WT genome. This is due to the automatic annotation of the CB1002 WT genome, which identified only 34% of the gene products and assigned an unknown function to the remaining 66% of the genes. Manually annotating and curating the CB1002 genome may allow for a better analysis of our data. Another limitation is that the experimental data and analysis are limited to the exponential growth phase of bacterial cultures in a rich liquid medium. Although we confirmed previous experimental data, this descriptive *in vitro* work does not account for interactions with the gut microbiota and requires further functional validation through additional experimental studies. One possible way to overcome this limitation would be to culture human gut microbiota *in vitro* using devices such as the Shime or Mipro systems and then analyze how the composition and functions of the microbiota change upon exposure to *C. butyricum* strains.

### Conclusion

*C. butyricum* is an intestinal commensal bacterium that has been observed to behave similarly to opportunistic pathogens that cause NEC. However, no classical repertoire of virulence genes has yet been identified that would allow for the unambiguous identification of pathogenic strains. In this study, we identified differential gene expression in an avirulent *hbd* mutant strain, which suggests that the bacterial cellular processes are reprogrammed to compensate for the consequences of the *hbd* deletion. These changes were consistent with different levels of various metabolites correlated with gene expression and protein interaction networks. If these cellular processes are indeed associated with the biological activities of *C. butyricum*, they may be implicated in its pathogenesis. Experimental data showed that the *hbd* deletion had no effect on *in vitro* inflammatory or oxidative activity when cellular models were exposed to the mutant strain. These results suggest that these processes may not contribute to the development of NEC-like lesions observed in an animal model of NEC. In conclusion, we identified several genes in *C. butyricum* that are promising targets for further study, particularly in the context of NEC pathogenesis. Generating mutants and in-depth characterizing those *in vitro* and *in vivo* settings will facilitate identifying the critical bottlenecks hindering invasion and survival. This will allow us to assess immune susceptibility and evaluate the enteropathogenicity of selected mutants in an NEC animal model. This will pave the way for validating new hypotheses about the role of *C. butyricum* and other related species as potential pathogens.

## Data Availability

The raw RNA-Seq data can be accessed via the ENA BioProject PRJEB81161. The CB1002 genome is available in the European Nucleotide Archive database under the accession number PRJEB81418.

## SUPPLEMENTAL MATERIAL

Supplemental material aem.01106-25-s0001.pdf Figures S1 to S4; Tables S1 and S2.

ASM does not own the copyrights to Supplemental Material that may be linked to, or accessed through, an article. The authors have granted ASM a non-exclusive, world-wide license to publish the Supplemental Material files. Please contact the corresponding author directly for reuse.
