# Supplementary material for: Transcriptome, metabolome, and inflammatory and oxidative properties of Clostridium butyricum CB1002 clinical strain and its isogenic mutant Δhbd
Source: Appl Environ Microbiol. 2026 Feb 23;92(3):e01106-25. doi: 10.1128/aem.01106-25 (PMC12997760; doi:10.1128/aem.01106-25)
Supplement: Supplemental material — Figures S1 to S4; Tables S1 and S2. [file aem.01106-25-s0001.pdf]

SUPPLEMENTARY MATERIALS

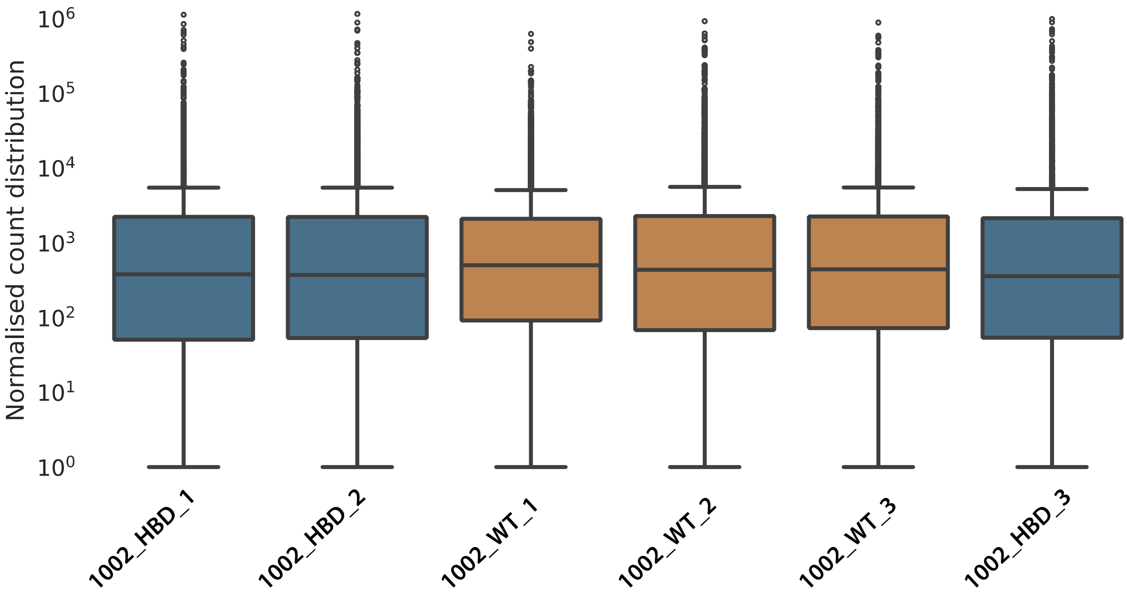

Figure S1. Boxplot of raw and normalized data contrasting CB1002 HBD (blue boxes) and CB1002 WT (orange boxes) triplicate samples.

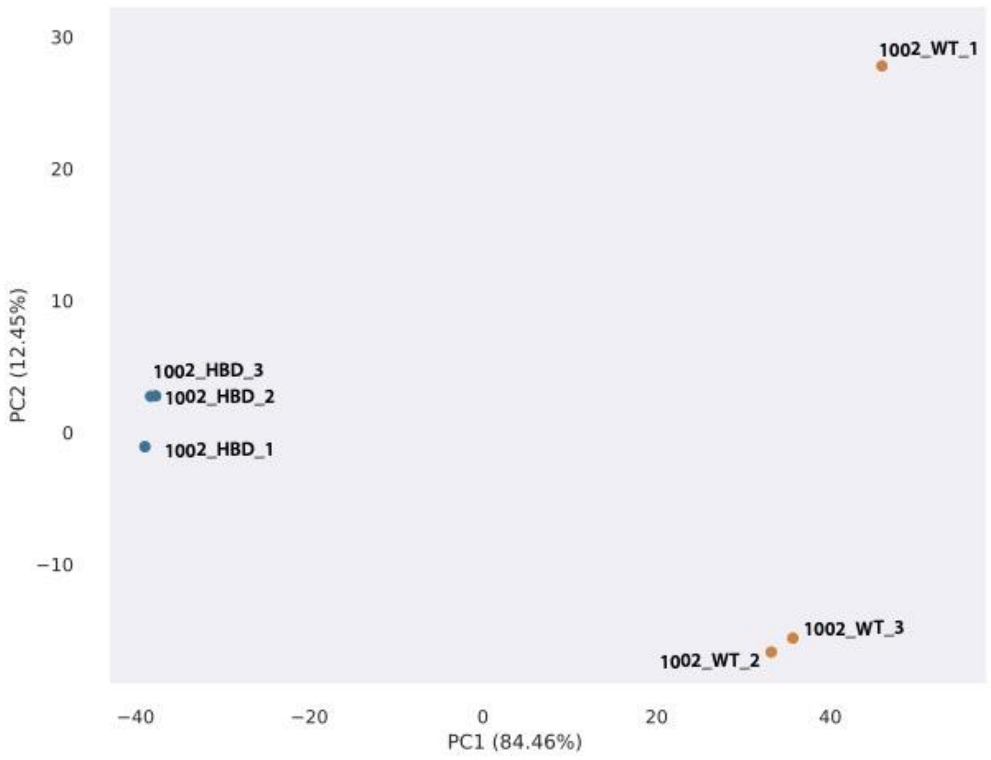

Figure S2. Principal component analysis plot showing the clustering of the conditions CB1002 HBD (blue) and CB1002 WT (orange) with 80% of the variance on component 1 showing the differences between the two conditions.

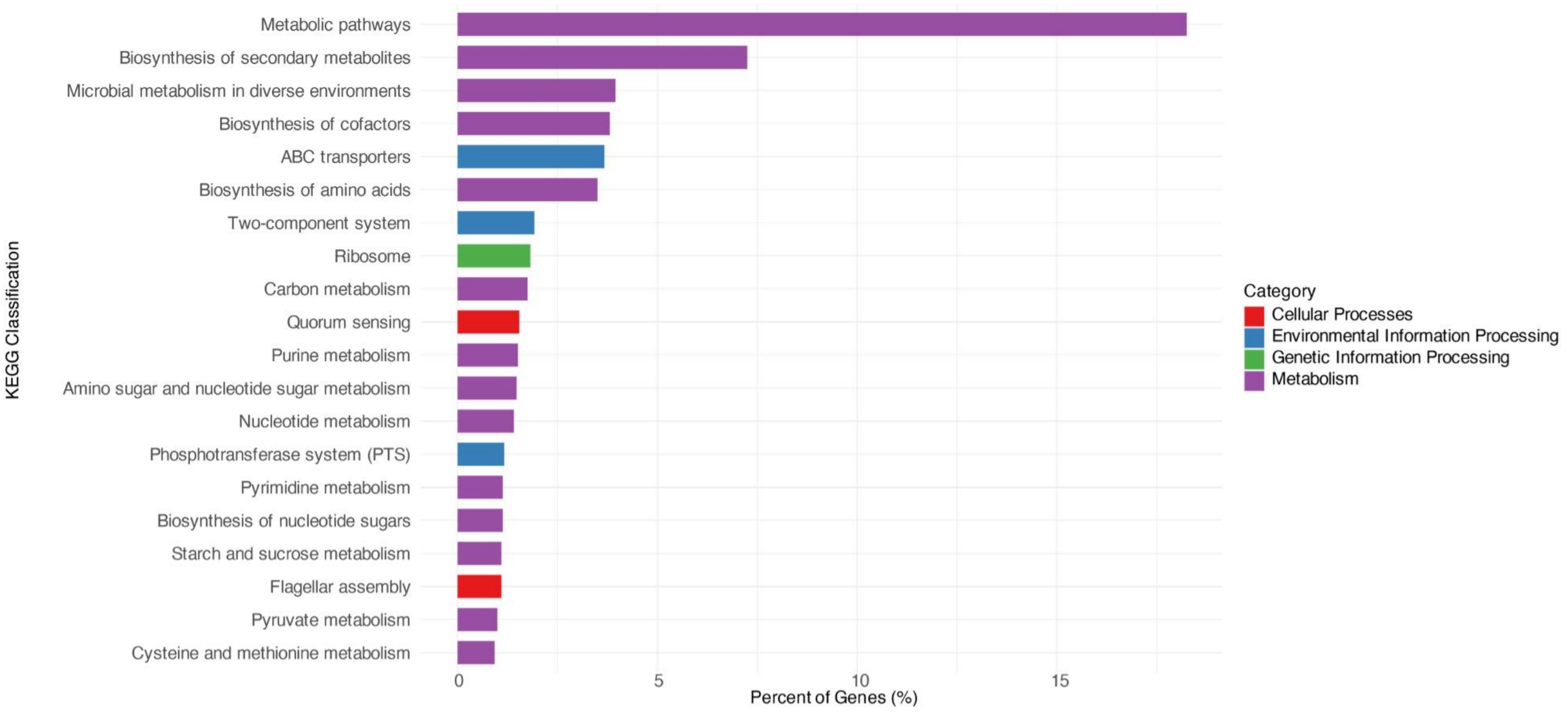

Figure S3. Top 20 enriched pathways according to the KEGG analysis, based on differentially expressed genes of CB1002 HBD vs CB1002 WT.

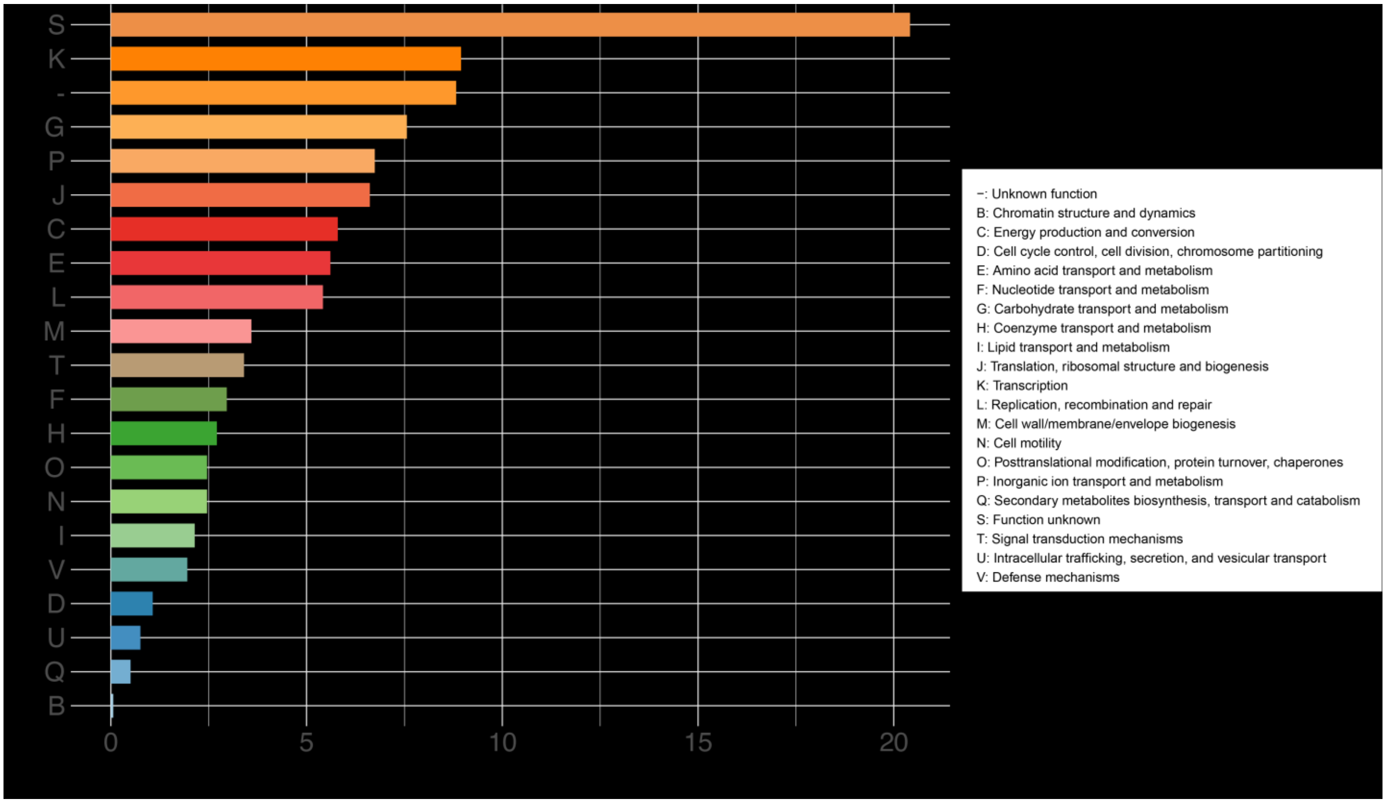

Figure S4. Clusters of COG database, based on differentially expressed genes of CB1002 HBD vs CB1002 WT.

Locus	Gene ID	log2Fold Change	Adjusted *p*-value	KEGG	Function	Class	Category	Subcategory
CBUT_v1_160022	*fliM*	-0.12	0.75	K02416	flagellar motor switch protein			
CBUT_v1_160023	*fliN*	-0.08	0.84	K02417	flagellar motor switch protein			
CBUT_v1_160024	*flgM*	-0.26	0.45	K02398	negative regulator of flagellin synthesis			
CBUT_v1_160026	*flgK*	-0.16	0.66	K02396	flagellar hook-associated protein 1			
CBUT_v1_160027	*flgL*	-0.09	0.81	K02397	flagellar hook-associated protein 3			
CBUT_v1_160032	*fliS*	0.04	0.9	K02422	flagellar secretion chaperone			
CBUT_v1_160033	*fliD*	-0.04	0.92	K02407	flagellar hook-associated protein			
CBUT_v1_160036	*fliC*	-0.01	0.98	K02406	flagellin protein			
CBUT_v1_160049	*fliC*	-0.05	0.86	K02406	flagellin protein			
CBUT_v1_160050	*flgB*	0.09	0.68	K02387	flagellar basal-body rod protein			
CBUT_v1_160051	*flgC*	0.22	0.42	K02388	flagellar basal-body rod protein			
CBUT_v1_160052	*fliE*	0.29	0.4	K02408	flagellar hook-basal body complex protein			
CBUT_v1_160053	*flif*	0.29	0.18	K02409	flagellar M-ring protein			
CBUT_v1_160054	*fliG*	0.41	0.12	K02410	flagellar motor switch protein			
CBUT_v1_160055	*fliH*	0.22	0.36	K02411	flagellar assembly protein			
CBUT_v1_160056	*fliI*	0.3	0.2	K02412	flagellum-specific ATP synthase			
CBUT_v1_160057	*fliJ*	0.47	0.15	K02413	flagellar protein	Flagellar assembly		
CBUT_v1_160058	*fliK*	0.28	0.29	K02414	flagellar hook-length control protein			
CBUT_v1_160059	*flgD*	0.12	0.7	K02389	flagellar basal-body rod modification protein			

22

CBUT_v1_160061	*flgE*	-0.06	0.87	K02390	flagellar hook protein			
CBUT_v1_160062	*flgE*	-0.11	0.77	K02390	flagellar hook protein			
CBUT_v1_120010	*motA*	-1.02	< 0.001	K02556	chemotaxis protein			
CBUT_v1_160064	*motA*	0.23	0.37	K02556	chemotaxis protein			
CBUT_v1_120011	*motB*	-0.86	< 0.001	K02557	chemotaxis protein			
CBUT_v1_160065	*motB*	0.15	0.63	K02557	chemotaxis protein			
CBUT_v1_160066	*fliL*	0.17	0.48	K02415	flagellar protein			
CBUT_v1_160067	*fliO, fliZ*	0.04	0.91	K02418	flagellar protein			
CBUT_v1_160068	*fliP*	0.24	0.41	K02419	flagellar biosynthesis protein			
CBUT_v1_160069	*fliQ*	0.26	0.41	K02420	flagellar biosynthesis protein			
CBUT_v1_160070	*fliR, flhB*	0.32	0.3	K13820	flagellar biosynthesis protein			
CBUT_v1_160071	*flhA*	0.37	0.19	K02400	flagellar biosynthesis protein			
CBUT_v1_160075	*fliA*	0.45	0.05	K02405	RNA polymerase sigma factor			
CBUT_v1_160078	*flgf*	0.21	0.38	K02391	flagellar basal-body rod protein			
CBUT_v1_160079	*flgG*	0.17	0.46	K02392	flagellar basal-body rod protein			
CBUT_v1_150033	*RpoD*	-0.02	0.96	K03086	RNA polymerase primary sigma factor			
CBUT_v1_170070	*RpoN*	-0.32	0.32	K03092	RNA polymerase primary sigma factor			
CBUT_v1_160014	*cheW*	-0.19	0.55	K03408	purine-binding chemotaxis protein			
CBUT_v1_160021	*cheW*	-0.31	0.27	K03408	purine-binding chemotaxis protein			Cell motility
CBUT_v1_160015	*cheD*	0.21	0.41	K03411	chemotaxis protein			
CBUT_v1_160016	*cheB*	0.32	0.25	K03412	chemotaxis protein			
CBUT_v1_160017	*cheR*	0.3	0.29	K00575	chemotaxis protein methyltransferase			

CBUT_v1_160018	*cheA*	0.07	0.83	K03407	sensor kinase				
CBUT_v1_160019	*chec*	0.01	0.98	K02406	chemotaxis protein				
CBUT_v1_130478	*cheY*	-1.59	< 0.001	K03413	chemotaxis protein				
CBUT_v1_160020	*cheY*	-0.09	0.77	K03413	chemotaxis protein				
CBUT_v1_190141	*cheV*	0.48	< 0.05	K03415	chemotaxis protein				
CBUT_v1_160022	*fliM*	-0.12	0.75	K02416	flagellar motor switch protein				
CBUT_v1_160023	*fliN*	-0.08	0.84	K02417	flagellar motor switch protein				
CBUT_v1_160054	*fliG*	0.41	0.12	K02410	flagellar motor switch protein				
CBUT_v1_130876	*mglB*	-0.06	0.88	K10540	methyl-galactoside transport system substrate-binding protein				
CBUT_v1_190539	*mglB*	-0.05	0.9	K10540	methyl-galactoside transport system substrate-binding protein	Bacterial chemotaxis			
CBUT_v1_230011	*mglB*	-0.31	0.5	K10540	methyl-galactoside transport system substrate-binding protein				
CBUT_v1_130957	*rbsB*	-3.59	< 0.001	K10439	ribose transport system substrate-binding protein				
CBUT_v1_180099	*rbsB*	0.44	0.32	K10439	ribose transport system substrate-binding protein				
CBUT_v1_130004	*mcp*	-0.22	0.38	K03406	methyl-accepting chemotaxis protein				
CBUT_v1_130921	*mcp*	4.55	< 0.001	K03406	methyl-accepting chemotaxis protein				
CBUT_v1_130962	*mcp*	1.07	< 0.001	K03406	methyl-accepting chemotaxis protein				
CBUT_v1_140076	*mcp*	-3.05	< 0.001	K03406	methyl-accepting chemotaxis protein				
CBUT_v1_180079	*mcp*	0.13	0.66	K03406	methyl-accepting chemotaxis protein				

CBUT_v1_190458	*mcp*	-0.93	< 0.001	K03406	methyl-accepting chemotaxis protein			
CBUT_v1_190541	*mcp*	2.14	< 0.001	K03406	methyl-accepting chemotaxis protein			
CBUT_v1_230026	*mcp*	0.3	0.39	K03406	methyl-accepting chemotaxis protein			
CBUT_v1_230060	*mcp*	-0.48	0.24	K03406	methyl-accepting chemotaxis protein			
CBUT_v1_280039	*mcp*	3.09	< 0.001	K03406	methyl-accepting chemotaxis protein			
CBUT_v1_290003	*mcp*	1.45	< 0.001	K03406	methyl-accepting chemotaxis protein			
CBUT_v1_290084	*mcp*	-0.32	0.2	K03406	methyl-accepting chemotaxis protein			
CBUT_v1_300126	*mcp*	-2.46	< 0.001	K03406	methyl-accepting chemotaxis protein			
CBUT_v1_300127	*mcp*	3.51	< 0.001	K03406	methyl-accepting chemotaxis protein		Cellular processes	
CBUT_v1_300136	*mcp*	1.22	< 0.01	K03406	methyl-accepting chemotaxis protein			
CBUT_v1_300161	*mcp*	-0.11	0.74	K03406	methyl-accepting chemotaxis protein			
CBUT_v1_370011	*mcp*	0.2	0.53	K03406	methyl-accepting chemotaxis protein			
CBUT_v1_120010	*motA*	-1.02	< 0.001	K02556	chemotaxis protein			
CBUT_v1_160064	*motA*	0.23	0.37	K02556	chemotaxis protein			
CBUT_v1_120011	*motB*	-0.86	< 0.001	K02557	chemotaxis protein			
CBUT_v1_160065	*motB*	0.15	0.63	K02557	chemotaxis protein			
CBUT_v1_130816	*aroF, aroG, aroH*	-1	< 0.01	K01626	3-deoxy-7-phosphoheptulonate synthase			
CBUT_v1_130831	*trpE*	-0.05	0.9	K01657	anthranilate synthase component I			

CBUT_v1_130830	*trpG*	0.01	0.98	K01658	anthranilate synthase component II			
CBUT_v1_190291	*livF*	0.09	0.82	K01996	branched-chain amino acid transport system ATP-binding protein			
CBUT_v1_190292	*livG*	0.08	0.84	K01995	branched-chain amino acid transport system ATP-binding protein			
CBUT_v1_190293	*livM*	0.2	0.63	K01998	branched-chain amino acid transport system permease protein			
CBUT_v1_190294	*livH*	0.57	0.28	K01997	branched-chain amino acid transport system permease protein			
CBUT_v1_300029	*ddpD*	-0.12	0.75	K02031	peptide/nickel transport system ATP-binding protein			
CBUT_v1_130458	*ddpB*	1.52	< 0.001	K02033	peptide/nickel transport system permease protein			
CBUT_v1_300031	*ddpB*	-0.55	0.16	K02033	peptide/nickel transport system permease protein			
CBUT_v1_130459	*ddpC*	1.8	< 0.001	K02034	peptide/nickel transport system permease protein			
CBUT_v1_300030	*ddpC*	0.07	0.87	K02034	peptide/nickel transport system permease protein			
CBUT_v1_130460	*ddpA*	1.66	< 0.001	K02035	peptide/nickel transport system substrate-binding protein			
CBUT_v1_300032	*ddpA*	-0.36	0.14	K02035	peptide/nickel transport system substrate-binding protein			
CBUT_v1_130462	*ddpF*	1.14	< 0.01	K02032	peptide/nickel transport system ATP-binding protein			

CBUT_v1_300028	*ddpF*	-0.12	0.71	K02032	peptide/nickel transport system ATP-binding protein			
CBUT_v1_250091	*ydcS*	0.3	0.22	K02055	putative spermidine/putrescine transport system substrate-binding protein			
CBUT_v1_170175	*secA*	0.12	0.62	K03070	preprotein translocase subunit			
CBUT_v1_50033	*secE*	0	1	K03073	preprotein translocase subunit			
CBUT_v1_170060	*secG*	-0.64	< 0.05	K03075	preprotein translocase subunit			
CBUT_v1_50066	*secY*	-0.08	0.85	K03076	preprotein translocase subunit			
CBUT_v1_130312	*ffh*	0.8	< 0.01	K03106	signal recognition particle subunit			
CBUT_v1_130310	*ftsY*	0.18	0.5	K03110	fused signal recognition particle receptor			
CBUT_v1_190523	*yajC*	0.11	0.72	K03210	preprotein translocase subunit			
CBUT_v1_380065	*yidC*	0.36	0.06	K03217	YidC/Oxa1 family membrane protein insertase			
CBUT_v1_130788	*hfq*	-0.31	0.36	K03666	host factor-I protein			
CBUT_v1_280027	*luxS*	-0.19	0.4	K07173	S-ribosylhomocysteine lyase	Quorum sensing		
CBUT_v1_170037	*kdpE*	-0.43	0.12	K07667	two-component system. OmpR family. KDP operon response regulator			
CBUT_v1_290131	*kdpE*	0.74	< 0.001	K07667	two-component system. OmpR family. KDP operon response regulator			

CBUT_v1_190322	*spo0A*	-1.79	< 0.001	K07699	two-component system. response regulator. stage 0 sporulation protein A			
CBUT_v1_130969	*agrC*	-3.62	< 0.001	K07706	two-component system. LytTR family. sensor histidine kinase			
CBUT_v1_230080	*agrC*	-2.56	< 0.001	K07706	two-component system. LytTR family. sensor histidine kinase			
CBUT_v1_230081	*agrC*	-2.82	< 0.001	K07706	two-component system. LytTR family. sensor histidine kinase			
CBUT_v1_130968	*agrA*	-2.78	< 0.001	K07707	two-component system. LytTR family. response regulator			
CBUT_v1_130088	*agrB*	-1.11	< 0.001	K07813	accessory gene regulator B			
CBUT_v1_130971	*agrB*	-2.96	< 0.001	K07813	accessory gene regulator B			
CBUT_v1_190534	*agrB*	-2.98	< 0.001	K07813	accessory gene regulator B			Cellular community
CBUT_v1_130541	*ydcZ*	1.1	< 0.01	K09936	bacterial transporter family-2 protein			
CBUT_v1_130542	*ydcZ*	1.08	< 0.01	K09936	bacterial transporter family-2 protein			
CBUT_v1_380028	*ydcZ*	-0.89	< 0.05	K09936	bacterial transporter family-2 protein			
CBUT_v1_130453	*oppF*	-2.94	< 0.001	K10823	oligopeptide transport system ATP-binding protein			
CBUT_v1_190508	*crp*	0.31	0.19	K10914	CRP/FNR family transcriptional regulator. cyclic AMP receptor protein			
CBUT_v1_360039	*crp*	0.08	0.84	K10914	CRP/FNR family transcriptional regulator. cyclic AMP receptor protein			

CBUT_v1_130779	*slo*	-1.11	< 0.001	K11031	thiol-activated cytolysin			
CBUT_v1_130367	*ribD*	-0.37	0.38	K11752	diaminohydroxyphosphoribosylaminopyrimidine deaminase / 5-amino-6-(5-phosphoribosylamino) uracil reductase			
CBUT_v1_140074	*ciaH*	0.22	0.24	K14982	two-component system. OmpR family. sensor histidine kinase			
CBUT_v1_140075	*ciaR*	0.34	0.11	K14983	two-component system. OmpR family. response regulator			
CBUT_v1_130454	*oppA*	-3.35	< 0.001	K15580	oligopeptide transport system permease protein			
CBUT_v1_50225	*oppA*	0.4	0.13	K15580	oligopeptide transport system permease protein			
CBUT_v1_130450	*oppB*	-3.31	< 0.001	K15581	oligopeptide transport system permease protein			
CBUT_v1_130451	*oppC*	-3.29	< 0.001	K15582	oligopeptide transport system permease protein			
CBUT_v1_130452	*oppD*	-3.28	< 0.001	K15583	oligopeptide transport system ATP-binding protein			
CBUT_v1_190068	*nisK*	0.34	0.22	K20487	two-component system. OmpR family. lantibiotic biosynthesis sensor histidine kinase			
CBUT_v1_190069	*nisR*	0.12	0.75	K20488	two-component system. OmpR family. lantibiotic biosynthesis response regulator			
CBUT_v1_190070	*nisG*	-0.14	0.71	K20492	lantibiotic transport system permease protein			
CBUT_v1_190071	*nisE*	0.12	0.76	K20491	lantibiotic transport system permease protein			

CBUT_v1_190072	*nisF*	0.01	0.98	K20490	lantibiotic transport system ATP-binding protein			
CBUT_v1_150042	*glp*	-3.49	< 0.001	K00688	glycogen phosphorylase	Biofilm formation		
CBUT_v1_50148	*glp*	-4.07	< 0.001	K00688	glycogen phosphorylase			
CBUT_v1_90005	*glgA*	-4.7	< 0.001	K00703	glycogen (starch) synthase			
CBUT_v1_90001	*glgD*	-4.13	< 0.001	K00975	Glycogen biosynthesis protein GlgD. glucose-1-phosphate adenylyltransferase family			
CBUT_v1_90002	*glgC*	-4.3	< 0.001	K00975	Glycogen biosynthesis protein GlgD. glucose-1-phosphate adenylyltransferase family			
CBUT_v1_160024	*flgM*	-0.26	0.45	K02398	negative regulator of flagellin synthesis			
CBUT_v1_160075	*fliA*	0.45	0.05	K02405	RNA polymerase sigma factor			
CBUT_v1_100001	*crr*	0.01	0.97	K02777	sugar PTS system EIIA component			
CBUT_v1_240087	*crr*	-4.5	< 0.001	K02777	sugar PTS system EIIA component			
CBUT_v1_160029	*csrA*	0.01	0.99	K03563	carbon storage regulator			
CBUT_v1_280027	*luxS*	-0.19	0.4	K07173	S-ribosylhomocysteine lyase			
CBUT_v1_190508	*crp*	0.31	0.19	K10914	CRP/FNR family transcriptional regulator. cyclic AMP receptor protein			
CBUT_v1_360039	*crp*	0.08	0.84	K10914	CRP/FNR family transcriptional regulator. cyclic AMP receptor protein			
CBUT_v1_130831	*trpE*	-0.05	0.9	K01657	anthranilate synthase component I			

CBUT_v1_130830	*trpG*	0.01	0.98	K01658	anthranilate synthase component II			
CBUT_v1_130788	*hfq*	-0.31	0.36	K03666	host factor-I protein			
CBUT_v1_170070	*rpoN*	-0.32	0.32	K03092	RNA polymerase sigma-54 factor			
CBUT_v1_130972	*cysE*	0.14	0.72	K00640	serine O-acetyltransferase			
CBUT_v1_170082	*cysE*	4.83	< 0.001	K00640	serine O-acetyltransferase			
CBUT_v1_170199	*wecB*	0.8	< 0.01	K01791	UDP-N-acetylglucosamine 2-epimerase (non-hydrolysing)			
CBUT_v1_170175	*secA*	0.12	0.62	K03070	preprotein translocase subunit	Bacterial secretion system	Environmental information processing	Membrane transport
CBUT_v1_190520	*secD*	-0.22	0.5	K03072	preprotein translocase subunit			
CBUT_v1_50033	*secE*	0	1	K03073	preprotein translocase subunit			
CBUT_v1_190519	*secF*	-0.11	0.74	K03074	preprotein translocase subunit			
CBUT_v1_170060	*secG*	-0.64	< 0.05	K03075	preprotein translocase subunit			
CBUT_v1_50066	*secY*	-0.08	0.85	K03076	preprotein translocase subunit			
CBUT_v1_130312	*ffh*	0.8	< 0.01	K03106	signal recognition particle subunit			
CBUT_v1_130310	*ftsY*	0.18	0.5	K03110	fused signal recognition particle receptor			
CBUT_v1_390001	*virD*	-0.54	< 0.05	K03205	type IV secretion system protein			
CBUT_v1_190523	*yajC*	0.11	0.72	K03210	preprotein translocase subunit			
CBUT_v1_380065	*yidC*	0.36	0.06	K03217	YidC family membrane protein insertase			
CBUT_v1_130972	*cysE*	0.14	0.72	K00640	serine O-acetyltransferase	Exopolysaccharide biosynthesis		
CBUT_v1_170082	*cysE*	4.83	< 0.001	K00640	serine O-acetyltransferase			

CBUT_v1_290175	*algI*	0.33	0.09	K19294	alginate O-acetyltransferase complex protein			
CBUT_v1_170114	*tagT, tagU, tagV*	-0.09	0.75	K01005	polyisoprenyl-teichoic acid--peptidoglycan teichoic acid transferase	Teichoic acid biosynthesis		
CBUT_v1_190462	*tagT, tagU, tagV*	-0.07	0.84	K01005	polyisoprenyl-teichoic acid--peptidoglycan teichoic acid transferase			
CBUT_v1_170008	*dltC*	-0.48	0.12	K14188	D-alanine--poly(phosphoribitol) ligase subunit 2			
CBUT_v1_170009	*dltB*	-0.21	0.4	K03739	membrane protein involved in D-alanine export			
CBUT_v1_170010	*dltA*	-0.33	0.19	K03367	D-alanine--poly(phosphoribitol) ligase subunit 1			
CBUT_v1_170012	*dltD*	-0.48	0.09	K03740	D-alanine transfer protein			
CBUT_v1_170209	*ugtP*	-0.29	0.28	K03429	processive 1.2-diacylglycerol beta-glucosyltransferase			
CBUT_v1_130124	*tagA*	0.37	0.14	K05946	N-acetylglucosaminyldiphosphoundecaprenol N-acetyl-beta-D-mannosaminyltransferase			
CBUT_v1_130136	*bacA*	0.22	0.49	K06153	undecaprenyl-diphosphatase			
CBUT_v1_130392	*bacA*	0.01	0.97	K06153	undecaprenyl-diphosphatase			
CBUT_v1_300101	*licD*	0.11	0.61	K07271	lipopolysaccharide cholinephosphotransferase			
CBUT_v1_130646	*ltaS*	0.44	0.09	K19005	lipoteichoic acid synthase			
CBUT_v1_190391	*bcrC*	0.5	< 0.01	K19302	undecaprenyl-diphosphatase			

CBUT_v1_280016	*bcrC*	0.6	< 0.01	K19302	undecaprenyl-diphosphatase			
CBUT_v1_290152	*murB*	0.27	0.2	K00075	UDP-N-acetylmuramate dehydrogenase			
CBUT_v1_170187	*murA*	-1.72	< 0.001	K00790	UDP-N-acetylglucosamine 1-carboxyvinyltransferase			
CBUT_v1_380025	*murA*	0.08	0.84	K00790	UDP-N-acetylglucosamine 1-carboxyvinyltransferase			
CBUT_v1_130329	*uppS*	-0.06	0.87	K00806	undecaprenyl diphosphate synthase			
CBUT_v1_250102	*uppS*	-0.37	0.19	K00806	undecaprenyl diphosphate synthase			
CBUT_v1_190372	*mraY*	0.29	0.17	K01000	phospho-N-acetylmuramoyl-pentapeptide-transferase			
CBUT_v1_170078	*ddl*	0.55	< 0.001	K01921	D-alanine-D-alanine ligase			
CBUT_v1_140033	*murC*	0.04	0.88	K01924	UDP-N-acetylmuramate--alanine ligase		Metabolism	Glycan biosynthesis and metabolism
CBUT_v1_140002	*murD*	-0.41	0.22	K01925	UDP-N-acetylmuramoylalanine--D-glutamate ligase			
CBUT_v1_190374	*murE*	0.29	0.17	K01928	UDP-N-acetylmuramoyl-L-alanyl-D-glutamate--2.6-diaminopimelate ligase			
CBUT_v1_190373	*murF*	0.11	0.61	K01929	UDP-N-acetylmuramoyl-tripeptide--D-alanyl-D-alanine ligase			
CBUT_v1_160005	*murG*	-0.27	0.4	K02563	UDP-N-acetylglucosamine--N-acetylmuramyl-(pentapeptide) pyrophosphoryl-undecaprenol N-acetylglucosamine transferase			
CBUT_v1_290154	*pbpA*	0.14	0.48	K05364	penicillin-binding protein A			

CBUT_v1_130043	*mrcA*	0.2	0.23	K05366	penicillin-binding protein 1A	Peptidoglycan biosynthesis		
CBUT_v1_170136	*mdra*	-0.09	0.7	K05515	penicillin-binding protein 2			
CBUT_v1_130136	*bacA*	0.22	0.49	K06153	undecaprenyl-diphosphatase			
CBUT_v1_130392	*bacA*	0.01	0.97	K06153	undecaprenyl-diphosphatase			
CBUT_v1_130108	*gatD*	0.15	0.46	K07009	lipid II isoglutaminyl synthase (glutamine-hydrolysing)			
CBUT_v1_130106	*dacC, dacA, dacD*	0.34	0.16	K07258	serine-type D-Ala-D-Ala carboxypeptidase (penicillin-binding protein 5/6)			
CBUT_v1_130544	*dacC, dacA, dacD*	-0.4	0.25	K07258	serine-type D-Ala-D-Ala carboxypeptidase (penicillin-binding protein 5/6)			
CBUT_v1_130550	*dacC, dacA, dacD*	0.47	0.05	K07258	serine-type D-Ala-D-Ala carboxypeptidase (penicillin-binding protein 5/6)			
CBUT_v1_170112	*dacC, dacA, dacD*	-0.53	< 0.05	K07258	serine-type D-Ala-D-Ala carboxypeptidase (penicillin-binding protein 5/6)			
CBUT_v1_190134	*vanY*	0.02	0.97	K07260	zinc D-Ala-D-Ala carboxypeptidase			
CBUT_v1_190375	*spoVD*	-0.12	0.74	K08384	stage V sporulation protein D (sporulation-specific penicillin-binding protein)			
CBUT_v1_190376	*spoVD*	-0.32	0.16	K08384	stage V sporulation protein D (sporulation-specific penicillin-binding protein)			
CBUT_v1_190391	*bcrC*	0.5	< 0.01	K19302	undecaprenyl-diphosphatase			

CBUT_v1_280016	*bcrC*	0.6	< 0.01	K19302	undecaprenyl-diphosphatase			
CBUT_v1_130109	*murT*	0.14	0.56	K23393	lipid II isoglutaminyl synthase (glutamine-hydrolysing)			

Table S1. Other differentially expressed genes between CB1002 HBD and CB1002 WT, based on automatic annotation through MaGe MicroScope platform software (v3.17.3).

In House database annotations	Variable number	Experimental m/z	Experimental RT (min)	Monoisotopic Molecular Weight	Log2 Fold Change	KEGG ID	Class	Sub-class
Lactic acid	M89T227_2	89.02423	3.78	90.03151	0.69	C00256	Organic Acids and Derivatives	Hydroxy Acids and Derivatives
Phenol	M95T68	95.04905	1.14	94.04177	0.14	C00146	Aromatic Homomonocyclic Compounds	Phenols and Derivatives
Oxobutyric acid	M101T129	101.02435	2.15	102.03163	0.34	C00109	Organic Acids and Derivatives	Keto-Acids and Derivatives
Nicotinic acid	M122T144	122.02461	2.4	123.03189	0.67	C00253	Aromatic Heteromonocyclic Compounds	Pyridines and Derivatives
Benzoic acid	M123T68_2	123.04396	1.14	122.03668	0.14	C00180	Aromatic Homomonocyclic Compounds	Benzoic Acid and Derivatives
Leucine	M132T78_5	132.10174	1.3	131.09446	0.14	C00123/C01933	Amino Acids Peptides and Analogues	Amino Acids and Derivatives
Hydroxyphenylethanol	M137T100	137.06061	1.67	138.06789	0.74	C06044	Aromatic Homomonocyclic Compounds	Phenols and Derivatives
Glutamine	M145T430	145.06174	7.17	146.06902	1.99	C00819/C00064	Amino Acids Peptides and Analogues	Amino Acids and Derivatives
N-Acetylserine	M146T285	146.04569	4.75	147.05297	2.53	C00979/C00979	Amino Acids. Peptides	Amino Acids and Derivatives
Histidine	M154T399_1	154.06196	6.65	155.06924	0.2	C00135	Amino Acids Peptides and Analogues	Amino Acids and Derivatives
Phenyllactic acid	M165T99	165.05537	1.66	166.06265	0.3	C01479	Aromatic Homomonocyclic Compounds	Benzyl Alcohols and Derivatives
N-Acetylglutamic acid	M188T448	188.05645	7.47	189.06373	0.43	C00624/C00624	Amino Acids Peptides and Analogues	Amino Acids and Derivatives
Indolelactic acid	M204T146	204.06619	2.43	205.07347	0.86	C02043	Aromatic Heteropolycyclic Compounds	Indoles

Apigenin flavone	M271T457	271.0597	7.62	270.05242	3.14	C01477/C06563	Aromatic Heteropolycyclic Compounds	Flavonoids
Pelargonidin	M271T386	271.06037	6.43	270.05309	1.88	C05904	Aromatic Heteropolycyclic Compounds	Flavonoids
Naringenin	M273T457	273.07538	7.62	272.0681	4.15	C00509	Aromatic Heteropolycyclic Compounds	Flavonoids
Glycitein	M285T418	285.0756	6.96	284.06832	0.64	C14536	Aromatic Heteropolycyclic Compounds	Flavonoids
Hexadecanedioic acid	M285T78	285.20697	1.29	286.21425	1.19	C19615	Organic Acids and Derivatives	Carboxylic Acids and Derivatives
Hydroxyoctadecadienoic acid	M295T76	295.22763	1.26	296.23491	1.1	*NA*	*NA*	*NA*
Isobutyric acid	M87T141	87.04501	2.34	88.05229	-4.32	C02632/C00466	Organic Acids and Derivatives	Carboxylic Acids and Derivatives
Monomethyl succinate	M131T133	131.03534	2.21	132.04262	-0.74	C08645/C08645	Organic Acids and Derivatives	Carboxylic Acids and Derivatives
Monomethyl adipate	M159T92	159.06606	1.53	160.07334	-4.64	*NA*	Organic Acids and Derivatives	Carboxylic Acids and Derivatives
N8-Acetylspermidine	M188T46	188.17566	0.77	187.16838	-1.47	C01029	Organic Acids and Derivatives	Carboxylic Acids and Derivatives
N6-Acetyl-Lysine	M189T56	189.12331	0.93	188.11603	-0.47	C02727	Amino Acids Peptides and Analogues	Amino Acids and Derivatives
Biopterin	M238T74	238.0935	1.23	237.08622	-0.97	C06313	Aromatic Heteropolycyclic Compounds	Pteridines and Derivatives

Deoxyadenosine	M252T83	252.10923	1.39	251.10195	-0.38	C00559	Nucleosides. Nucleotides and Analogues	Purine Nucleosides and Analogues
Cyclic AMP	M330T59	330.05927	0.99	329.05199	-0.29	C00575	Nucleosides. Nucleotides and Analogues	Purine Nucleotides
Tetrasaccharides	M665T594	665.21316	9.89	666.22044	-0.64	C02052/C01613	Carbohydrates and Carbohydrate Conjugates	Tetrasaccharides

Table S2. List of 28 significantly differentially identified metabolites from non-targeted metabolomics of CB1002 HBD compared to CB1002 WT from the culture supernatant used for RNA-Seq analysis.
